# Circular RNA circBFAR promotes the progression of pancreatic ductal adenocarcinoma via the miR-34b-5p/MET/Akt axis

**DOI:** 10.1186/s12943-020-01196-4

**Published:** 2020-05-06

**Authors:** Xiaofeng Guo, Quanbo Zhou, Dan Su, Yuming Luo, Zhiqiang Fu, Leyi Huang, Zhiguo Li, Decan Jiang, Yao Kong, Zhihua Li, Rufu Chen, Changhao Chen

**Affiliations:** 1 Department of Pancreatobiliary Surgery, Sun Yat-sen Memorial Hospital, 107 Yanjiangxi Road, Yuexiu District, Guangzhou, Guangdong 510120 P. R. China; 2 Guangdong Provincial Key Laboratory of Malignant Tumor Epigenetics and Gene Regulation, Sun Yat-sen Memorial Hospital, State Key Laboratory of Oncology in South China, 107 Yanjiangxi Road, Yuexiu District, Guangzhou, Guangdong 510120 P. R. China; 3 Affiliated Huadu Hospital, Southern Medical University, 48 Xinhua Road, Huadu District, Guangzhou, Guangdong 510800 P. R. China; 4 Department of Ultrasound, Sun Yat-sen Memorial Hospital, 107 Yanjiangxi Road, Yuexiu District, Guangzhou, Guangdong 510120 P. R. China; 5 Department of Medical Oncology, Sun Yat-sen Memorial Hospital, 107 Yanjiangxi Road, Yuexiu District, Guangzhou, Guangdong 510120 P. R. China; 6 Department of General Surgery, Guangdong Provincial People’s Hospital, Guangdong Academy of Medical Sciences, 106 Zhongshan 2nd Road, Yuexiu District, Guangzhou, Guangdong 510080 P.R. China; 7 Department of Urology, Sun Yat-sen Memorial Hospital, 107 Yanjiangxi Road, Yuexiu District, Guangzhou, Guangdong 510120 P. R. China

**Keywords:** circBFAR, miR-34b-5p, MET, PI3K/Akt pathway, Pancreatic ductal adenocarcinoma

## Abstract

**Background:**

Accumulating evidence suggests that circular RNAs (circRNAs) are important participants in cancer progression. However, the biological processes and underlying mechanisms of circRNAs in pancreatic ductal adenocarcinoma (PDAC) are unclear.

**Method:**

CircRNAs were verified by Sanger sequencing. Colony formation, 5-Ethynyl-2′-deoxyuridine (EdU), and Transwell assays were performed to investigate the effect of circBFAR on the proliferation, invasion, and migration of PDAC cells in vitro. RNA pull-down assays were conducted to verify the binding of circBFAR with microRNA miR-34b-5p.

**Results:**

In the present study, we identified a novel circRNA (termed as circBFAR, hsa_circ_0009065) that was upregulated in a 208-case cohort of patients with PDAC. The ectopic expression of circBFAR correlated positively with the tumor-node-metastasis (TNM) stage and was related to poorer prognosis of patients with PDAC. Moreover, circBFAR knockdown dramatically inhibited the proliferation and motility of PDAC cells in vitro and their tumor-promoting and metastasis properties in in vivo models. Mechanistically, circBFAR upregulated mesenchymal-epithelial transition factor (MET) expression via sponging miR-34b-5p. Additionally, circBFAR overexpression increased the expression of MET and activated downstream phosphorylation of Akt (Ser 473) and further activated the MET/PI3K/Akt signaling pathway, which ultimately promoted the progression of PDAC cells. Importantly, application of MET inhibitors could significantly attenuate circBFAR-mediated tumorigenesis in vivo.

**Conclusions:**

Our findings showed that circBFAR plays an important role in the proliferation and metastasis of PDAC, which might be explored as a potential prognostic marker and therapeutic target for PDAC.

## Background

Pancreatic ductal adenocarcinoma (PDAC) is one of the deadliest cancers in developed countries and is likely to rise to second place within the next decade worldwide [1–3]. Despite survival being greatly improved in other cancers, the 5-year survival rate for PDAC remains < 3%, and the median survival time of patients with PDAC is usually less than 7.8 months in China [4, 5]. The highly aggressive phenotype, which is characterized by rapid invasion and high risk of recurrence and metastasis, is one of the most important causes of the high mortality in PDAC [6, 7]. Despite considerable advances in the prevention, diagnosis, and treatment of PDAC, no effective biomarkers or notably better therapeutic strategies have emerged [8, 9]. Thus, it is crucial to further explore the biological and molecular mechanism of PDAC progression and develop a molecule-oriented method for early diagnosis and targeted therapy.

Circular RNAs (circRNAs) are non-coding RNAs with a covalently closed loop structure and arise from the back-splicing of pre-mRNA transcripts [10, 11]. Unlike linear RNAs, circRNAs contain neither 5′-3′ polarities nor polyadenylated tails [12]. Moreover, as a novel type of endogenous non-coding RNA, circRNAs are widespread, conserved, stable, and tissue specific [11, 13]. Emerging evidence shows that circRNAs mainly function as a miRNA sponge to regulate the expression of the downstream target gene [14]. In addition, some circRNAs have been found to interact with RNA binding proteins (RBPs) or function as templates for protein translation [15, 16]. Moreover, previous studies have demonstrated that circRNAs exert impact on biological processes of cancers, including proliferation, migration, invasion, and apoptosis [17]. However, the potential correlation between circRNAs and PDAC progression and the underlying mechanism remains unclear.

The mesenchymal-epithelial transition factor (MET), identified as a pivotal tyrosine kinase, plays critical roles in biological processes such as cell proliferation, morphogenesis, survival, and the initialization and progression of cancer [18, 19]. Previous studies have demonstrated that MET was frequently overexpressed and played an important role in the progression of PDAC. For example, Neesse A.et al. found that MET is involved in the stromal biology of PDAC and promotes cancer progression [20]. Logan-Collins.et al. revealed that MET plays critical roles in chemotherapy and radiation resistant of PDAC [21]. Recently, increasing evidence has confirmed that circRNAs participate in tumorigenesis and the progression of a variety of cancers by regulating their downstream target genes [22]. Thus, exploring the association between circRNAs and MET is of great significance for the development of effective therapeutic strategies in PDAC.

In the present study, we identified an oncogenic circRNA generated from exon 2 of the BFAR gene, termed circBFAR, which was overexpressed in a 208-case cohort of patients with PDAC. We demonstrated that circBFAR was aberrantly upregulated in PDAC cells, induced the proliferation and invasiveness phenotype in vitro, and promoted tumorigenesis and metastasis in vivo*.* Moreover, circBFAR overexpression correlated positively with progression and was related to poorer prognosis of patients with PDAC. Importantly, we revealed that circBFAR sponged miR-34b-5p to upregulate MET expression and therefore promoted PDAC progression. Administration of a MET inhibitor could effectively attenuate circBFAR-mediated tumorigenicity of PDAC cells in vivo. Collectively, our study revealed that the circBFAR/miR-34b-5p/MET axis played a crucial role in PDAC progression and in particular, identified circBFAR as a potential biomarker and therapeutic target in PDAC.

## Methods

### Clinical ***specimens***

Fresh PDAC tissues and normal adjacent tissues (NATs) were collected from Sun Yat-sen Memorial Hospital from January 2014 to June 2018. Our research was approved by the Institutional Review Board of Sun Yat-sen Memorial Hospital, Sun Yat-sen University, and signed informed consent was obtained from each patient before participation in the research. Each sample was evaluated by professional pathologists. These samples were stored at − 80 °C until required. The information of patients’ background and characteristics is summarized in Additional file 1.

### RNase R treatment

Total RNA from cells was extracted using the Trizol reagent (Takara, Shiga, Japan) and subsequently divided into two aliquots: one was used for RNase R digestion and 2 μg of total RNA was mixed with buffer and incubated for 15 min at 37 °C in 3 U/mg RNase R (Epicentre Technologies, Madison, WI, USA); the other aliquot was the control, which was treated with 0.2 μl of DEPC water. GAPDH was used as the internal control [23].

### Biotin-labeled probe pull-down assay

Biotin-labeled oligonucleotide probes targeting junction sites of circBFAR were synthesized as previously described [24]. 1 × 10^7^ PDAC cells were harvested and fixed with 1% formaldehyde. The cells were then lysed for 15 min in lysis buffer [0.02 M Tris-HCl (pH 7.5), 0.1 M KCl, 5 mM MgCl_2_, 0.5 mM DTT, 0.5% NP-40, 60 U/ml RNase inhibitor (Promega), 1× protease inhibitor EDTA-free (05892791001, Sigma, USA)] and the supernatant was collected after centrifugation. At the same time, the biotinylated probe or oligo probe was incubated with streptavidin-coated magnetic beads (Invitrogen, Waltham, MA, USA) for 2 h. The circBFAR probe or oligo probe was then mixed with cell lysates at 4 °C overnight. The next day, beads were added to RNAiso Plus (Takara, Japan) to extract the RNA after they were washed with wash buffer (0.1%SDS, 1%Trition X-100, 2 mM EDTA, 20 mM Tris-HCl pH 8.0, 150 mM NaCl). Reverse transcription and quantitative real-time PCR (qRT-PCR) assays were performed using the TB Green Premix Ex Taq™ kit (Takara). The sequences of probes are listed in Additional file 2.

### Animal experiments

The subcutaneous xenograft models were conducted as previously described [25]. Severe combined immunodeficient (SCID) mice aged 4–6 weeks were randomized blindly. A total of 5 × 10^6^ cells containing the circBFAR knockdown vector or control vector were subcutaneously injected into right hind flank of the mice. The volume of the tumors was measured every 4 days. Four weeks later, the mice were sacrificed, and tumor tissues were excised, measured, and weighed. The tumor volume was calculated as follows: volume = (width^2^ × length)/2.

The tail vein injection models were conducted as previously described [26]. SCID mice were divided into two groups randomly. PANC-1 cells stably transfected with circBFAR knockdown plasmid or control plasmid were injected into the tail vein (2 × 10^6^ cells per mouse), respectively. Mice were sacrificed 6 weeks later. Lung tissues were resected, and metastatic foci were examined under the microscope and subject to hematoxylin and eosin (HE) staining. The lung metastatic fluorescence images were detected using an in vivo FX PRO system (BRUKER Corporation, Billerica, MA, USA).

### Statistical analysis

The statistical analyses were performed using SPSS version 19.0 (IBM Corp., Armonk, NY, USA) and GraphPad Prism version 8.0 (GraphPad Software, La Jolla, CA, USA). Survival analysis was performed using the Kaplan-Meier method, and the log-rank test was carried out to compare the survival curves. Student’s t-test or one-way analyses of variance (ANOVA) was used to compare group differences if they followed a normal distribution; otherwise, the nonparametric Mann-Whitney test was adopted. The relationship between the circBFAR and the clinicopathological parameters of the patients was analyzed using a Chi-squared test. All data were presented as the mean ± the standard deviation from at least three independent experiments, unless otherwise noted. All the tests were two-tailed and *P* < 0.05 indicated a statistically significant difference.

### Further applied methods

Additional microarray analysis; cell culture; actinomycin D assay; fluorescence in situ hybridization (FISH); circBFAR plasmid construction and stable transfection; oligonucleotide transfection; RNA extraction and qRT-PCR; colony formation; cell counting kit 8 (CCK)-8 assay; 5-Ethynyl-20-deoxyuridine (EdU) incorporation; wound healing, Transwell; subcellular fractionation, biotin-labeled miRNA capture; dual luciferase reporter assay; western blotting; and immunohistochemistry (IHC) are further described in Additional file 3.

## Results

### The identification and characteristics of circBFAR in PDAC cells

**Fig. 1:**
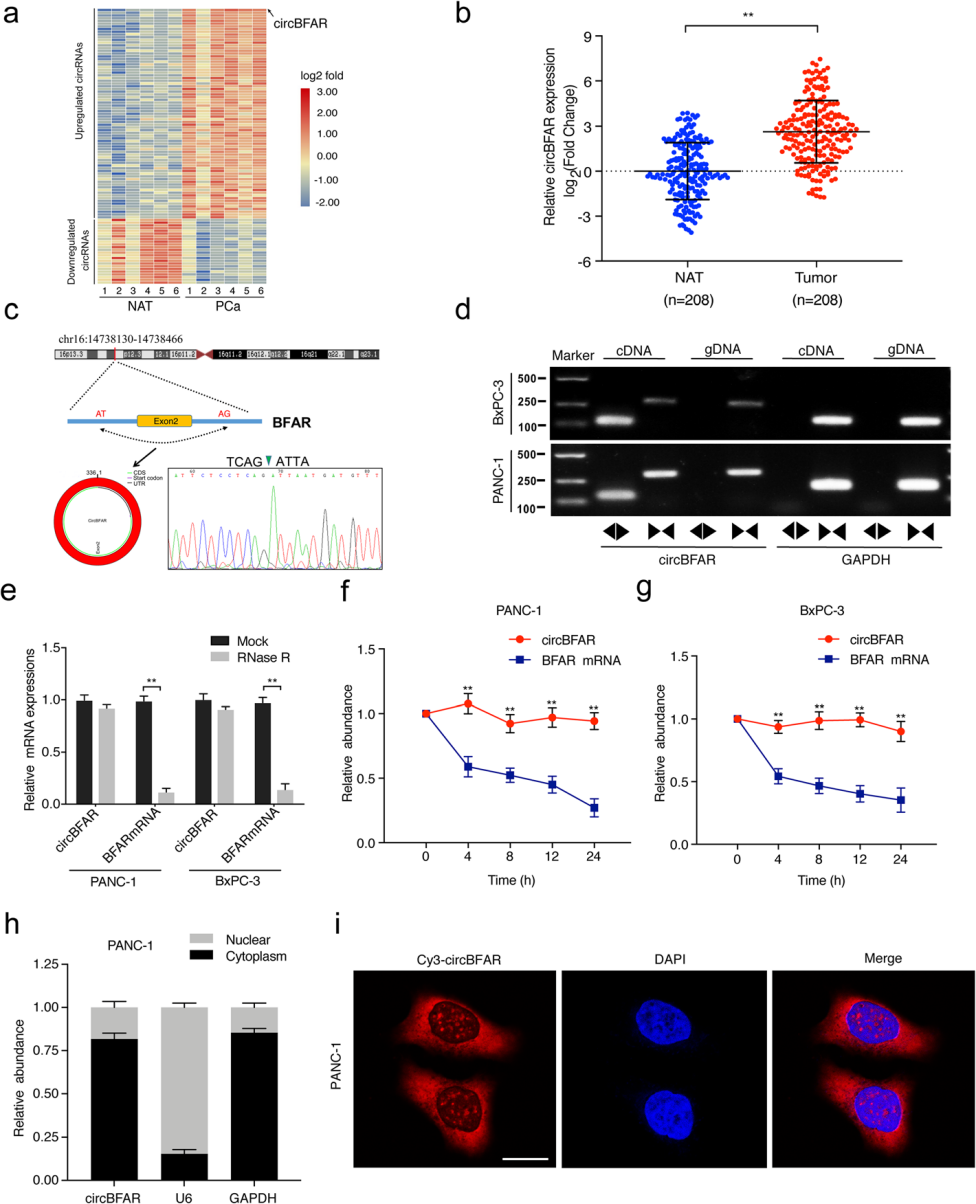
Identification and characterization of circBFAR in PDAC cells. **a** A heatmap showing the differentially expressed circRNAs in six pancreatic cancer tissues and corresponding NATs. The red and blue scales represent higher or lower expression levels, respectively. **b** qRT-PCR analysis of circBFAR in 208 paired PDAC and adjacent noncancerous tissues. The nonparametric Mann-Whitney U-test was used. **c** Schematic illustration showing the genomic loci of the BFAR gene and the circBFAR derived from exon 2 of BFAR. A green arrow indicates the “head-to-tail” splicing sites of circBFAR, which were validated by Sanger sequencing. **d** Combining PCR with an electrophoresis assay indicated the presence of circBFAR using divergent and convergent primers from cDNA or genomic DNA (gDNA) in PANC-1 and BxPC-3 cells. **e** qRT-PCR analysis for the resistance of circBFAR and linear BFAR to RNase R in PANC-1 and BxPC-3 cells. The mock treatment is the negative control. Two-tailed t-tests was used. **f**, **g** Actinomycin D assay to evaluate the stability of circBFAR and BFAR mRNA in PANC-1 and BxPC-3 cells. Two-tailed t-tests was used. **h** The location of circBFAR was confirmed using a subcellular fractionation assay and qRT-PCR data indicate that circBFAR is mainly located in the cytoplasm. **i** Representative FISH images showing the cellular localization of circBFAR. The circBFAR probe was labeled with Cy3 (red), nuclei were stained with DAPI (blue). The images were photographed at 1000X magnification. Scale bar = 10 μm. The error bars represent the standard deviations of three independent experiments. *P < 0.05, **P < 0.01

To identify critical circRNAs that contribute to PDAC progression, we first analyzed microarray data in six pairs of PDAC and matched NATs [27]. The analysis identified 77 circRNAs upregulated more than 2-fold (*p* < 0.05) (Fig. 1a). We selected top 20 candidate circRNAs according to their fold changes and searched for those, from which the parental genes highly expressed and correlated with prognosis in PDAC in TCGA database. Subsequently, hsa_circ_0003763, hsa_circ_0092314, hsa_circ_0060055 and hsa_circ_0009065 were selected for further validation in a cohort of 208-case of PDAC patients and only hsa_circ_0009065 (termed circBFAR) was significantly upregulated in PDAC tissues compared with that in matched NATs (Fig. 1b). We then focused on circBFAR for further study.

We investigated the structure of circBFAR based on the circBase database annotation. The results demonstrated that circBFAR is located at chromosome 16p13.12 (NM_016561) and is derived from exon 2 of the BFAR gene with a length of 336 nt (Fig. 1c). To further confirm the circular form of circBFAR, we designed two sets of primers: convergent primers and divergent primers, which were used to amplify BFAR mRNA and the circular form, circBFAR, respectively. Sanger sequencing confirmed that the PCR products amplified using the divergent primers contained the head-to-tail splicing site of circBFAR and the sequence was consistent with the circBase database annotation (Fig. 1c). Subsequently, the existence of circBFAR was confirmed using divergent primers PCR with different templates (Fig. 1d). Meanwhile, an RNase R digestion assay showed that circBFAR was resistant to RNase R treatment, which is a 3′ to 5′ exoribonuclease, while linear BFAR was significantly degraded after RNase R treatment, further confirming the circular form of circBFAR (Fig. 1e). Given that circRNAs are more stable than linear RNAs, we further analyzed the stability of circBFAR in PDAC cells. After treatment with actinomycin D, which was used to suppress RNA transcription, the half-life of the circBFAR transcript was significantly longer than that of linear BFAR, indicating that circBFAR was highly stable in PDAC cells (Fig. 1f-g).

Furthermore, we investigated the cellular localization of circBFAR in PDAC cells. Subcellular fractionation and FISH assays revealed that circBFAR is mainly distributed in the cytoplasm (Fig. 1h-i). Collectively, these results demonstrated that circBFAR, located in the cytoplasm of PDAC cell lines, as a highly stable circRNA.

### CircBFAR promotes the proliferation, migration, and invasion of PDAC cells in vitro

**Fig. 2:**
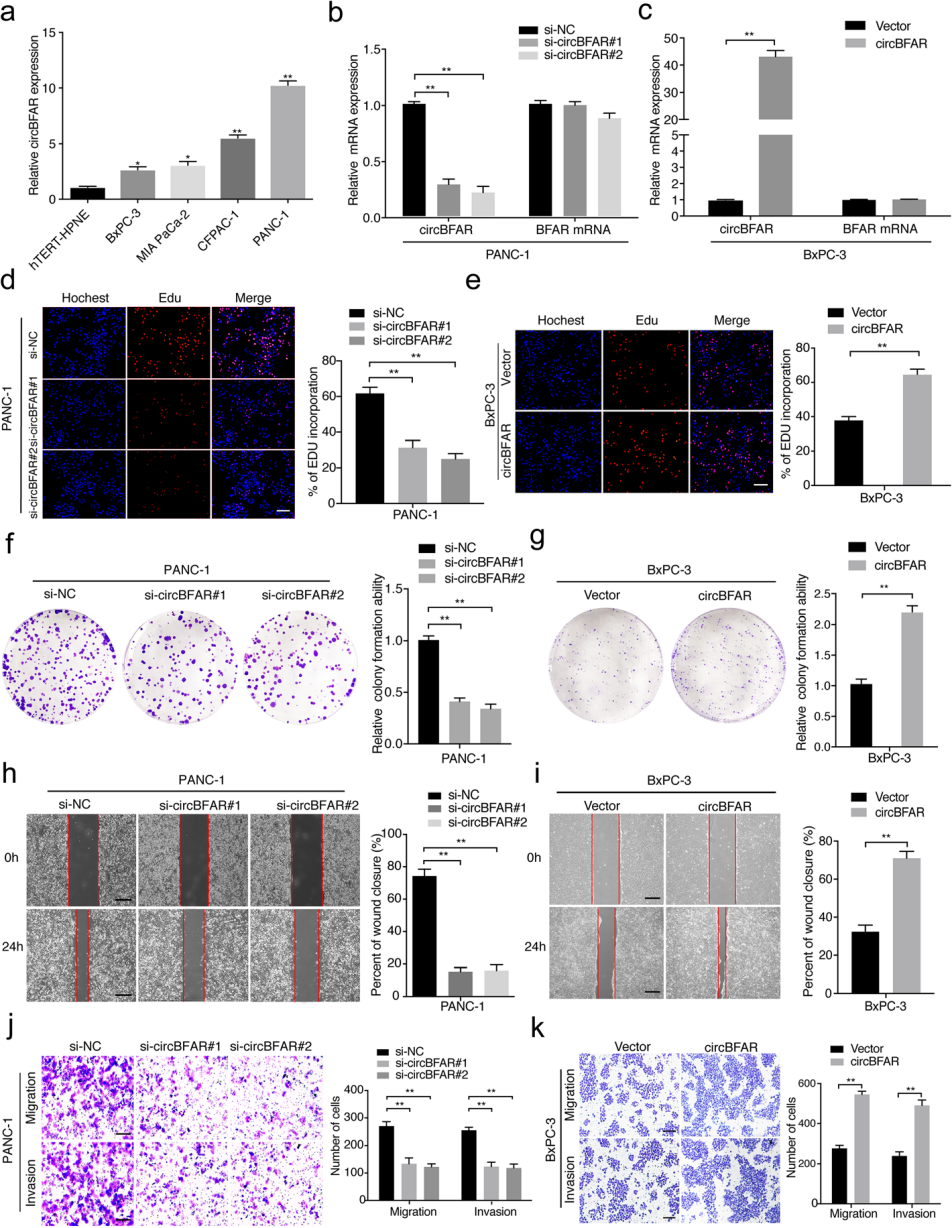
CircBFAR promotes the proliferation, migration, and invasion of PDAC cells in vitro. **a** qRT-PCR analysis for the expression of circBFAR in pancreatic epithelial cells (hTERT-HPNE) and PDAC cells (BxPC-3, MIA PaCa-2, CFPAC-1, and PANC-1). **b, c** The expression of circBFAR and BFAR mRNA was assessed by qRT-PCR in PANC-1 cells treated with an siRNA (**b**) and BxPC-3 cells transfected with the circBFAR plasmid (**c**) and control cells, as indicated. **d**, **e** EdU assays showing that knockdown of circBFAR inhibited the DNA synthesis of PANC-1 cells (**d**), while overexpression of circBFAR promoted DNA synthesis in BxPC-3 cells (**e**). The images were photographed at 100X magnification. Scale bar = 100 μm. **f**, **g** Colony formation assays to evaluate the cell proliferation ability after knocking down circBFAR in PANC-1 cells (**f**) and overexpressing circBFAR in BxPC-3 cells (**g**). **h**, **i** The migration capability of circBFAR was suppressed in PANC-1 cells treated with si-circBFAR#1 and si-circBFAR#2 (**h**), while migration was promoted in BxPC-3 cells transfected with the circBFAR plasmid, as determined using a wound healing assay (**i**). The images were photographed at 40X magnification. Scale bar = 200 μm. **j**, **k** The cell migration and invasion ability were measured using Transwell migration and Matrigel invasion assays after knocking down circBFAR in PANC-1 cells (**j**) and overexpression circBFAR in BxPC-3 cells (**k**). The images were photographed at 100X magnification. Scale bar = 100 μm. Statistical significance was assessed using two-tailed t-tests for two group comparison, and one-way ANOVA followed by Dunnett’s tests for multiple comparison. The error bars represent the standard deviations of three independent experiments. *P < 0.05, **P < 0.01

To assess the proliferation and aggressiveness of circBFAR in PDAC cells, gain-and loss-of-function assays were conducted. Firstly, we assessed the expression of circBFAR in different PDAC cells and normal pancreatic cell lines. CircBFAR was significantly upregulated in PDAC cells (BxPC-3, MIA PaCa-2, CFPAC-1, and PANC-1) compared with that in the normal pancreatic cell line hTERT-HPNE (Fig. 2a). We constructed two short interfering RNAs targeting the back-splice site of circBFAR to specifically downregulate the expression of circBFAR in PANC-1 and CFPAC-1 cells (Fig. 2b and Additional file 4: Figure S1a and S1b). Meanwhile, we performed ectopic expression of circBFAR using a circBFAR plasmid without affecting BFAR expression in BxPC-3 cells (Fig. 2c). EdU and colony formation assays showed that knockdown of circBFAR suppressed the proliferation of PDAC cells (Fig. 2d and f and Additional file 4: Figure S1c-f). Conversely, overexpression of circBFAR had the opposite effects on proliferation, indicating that circBFAR promotes the proliferation of PDAC cells (Fig. 2e and g).

Given that PDAC is more prone to metastasize, we further studied whether circBFAR affected the invasion and migration of PDAC cells. A wound-healing assay confirmed that the migration ability was significantly inhibited after knockdown of circBFAR in PDAC cells (Fig. 2h and Additional file 4: Figure S1g and S1h). Consistently, circBFAR knockdown also reduced the migration and invasion of PDAC cells in Transwell assays (Fig. 2j and Additional file 4: Figure S1i and S1j). Conversely, overexpression of circBFAR exhibited the opposite effects (Fig. 2i and k). Taken together, these findings suggested that circBFAR facilitates the proliferation, migration, and invasion of PDAC cells in vitro.

### CircBFAR promotes tumor growth and metastasis of PDAC in vivo

**Fig. 3:**
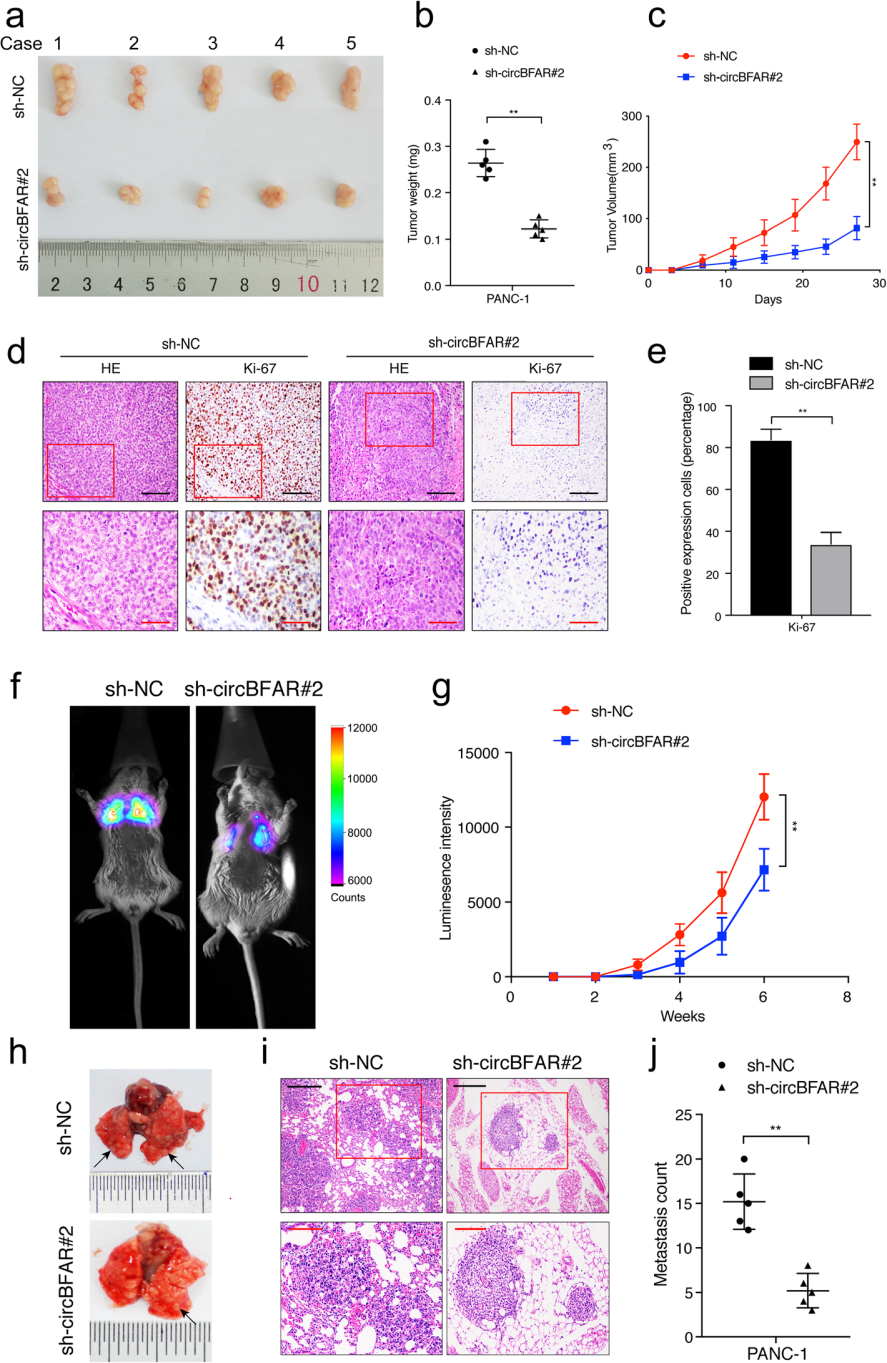
CircBFAR promotes tumor growth and metastasis of PDAC cells in vivo. **a** Representative images of subcutaneous xenograft tumors. **b**, **c** The tumor volume and weight dramatically decreased in sh-circBFAR#2 treated mice compared with those treated with the control shRNA. **d**, **e** Representative HE and IHC staining images of subcutaneous tumors revealed the relative protein levels of Ki-67 in different groups. The images were photographed at 200X (upper panel) or 400X (lower panel) magnification. Scale bar: black =100 μm; red =50 μm. **f**, **g** Representative IVIS images and analysis of luminescence intensity in lung in tail-vein injection model (*n* = 6 for each group). **h** Representative images of lung metastatic tumors. **i** HE staining of lung metastatic tumors. The images were photographed at 100X (upper panel) or 200X (lower panel) magnification. Scale bar: black =200 μm; red =100 μm. **j** The number of lung metastatic tumors decreased significantly in sh-circBFAR#2 treated mice. Statistical significance was assessed using two-tailed t-tests for two group comparison, and one-way ANOVA followed by Dunnett’s tests for multiple comparison. The error bars represent the standard deviations of three independent experiments. **P < 0.01

To determine whether circBFAR contributed to tumor growth of PDAC in vivo*,* a xenograft mouse model was constructed. We first analyzed the knockdown efficiency of sh-circBFAR transfection in PDAC cells. The results confirmed that the expression of circBFAR was significantly downregulated in PDAC cells stably transfected with sh-circBFAR (Additional file 5: Figure S2a). Subsequently, PANC-1 cells with stable knockdown of circBFAR or transformed with the control vector were subcutaneously injected into right hind flank of SCID mice. The results showed that knockdown of circBFAR inhibited tumor growth (Fig. 3a). Lower tumor weight and volume were observed in the circBFAR group compared with those in the control group (Fig. 3b-c). IHC staining revealed that Ki-67 levels were markedly reduced by knockdown of circBFAR (Fig. 3d-e).

We further analyzed the effect of circBFAR on the metastasis of PDAC in vivo via a tail vein injection model. Luciferase-labeled PANC-1 cells were injected into the tail vein of SCID mice. Knockdown of circBFAR reduced the fluorescence intensity in the lung compared with that of the control group, suggesting that circBFAR knockdown inhibited the metastasis of PDAC cells to the lung (Fig. 3f-g). Moreover, fewer lung metastatic foci were observed in the circBFAR knockdown group than in the control group (Fig. 3h-j). These results suggested that circBFAR promotes tumorigenesis and metastasis of PDAC in vivo.

### CircBFAR serves as a sponge for miR-34b-5p in PDAC cells

**Fig. 4:**
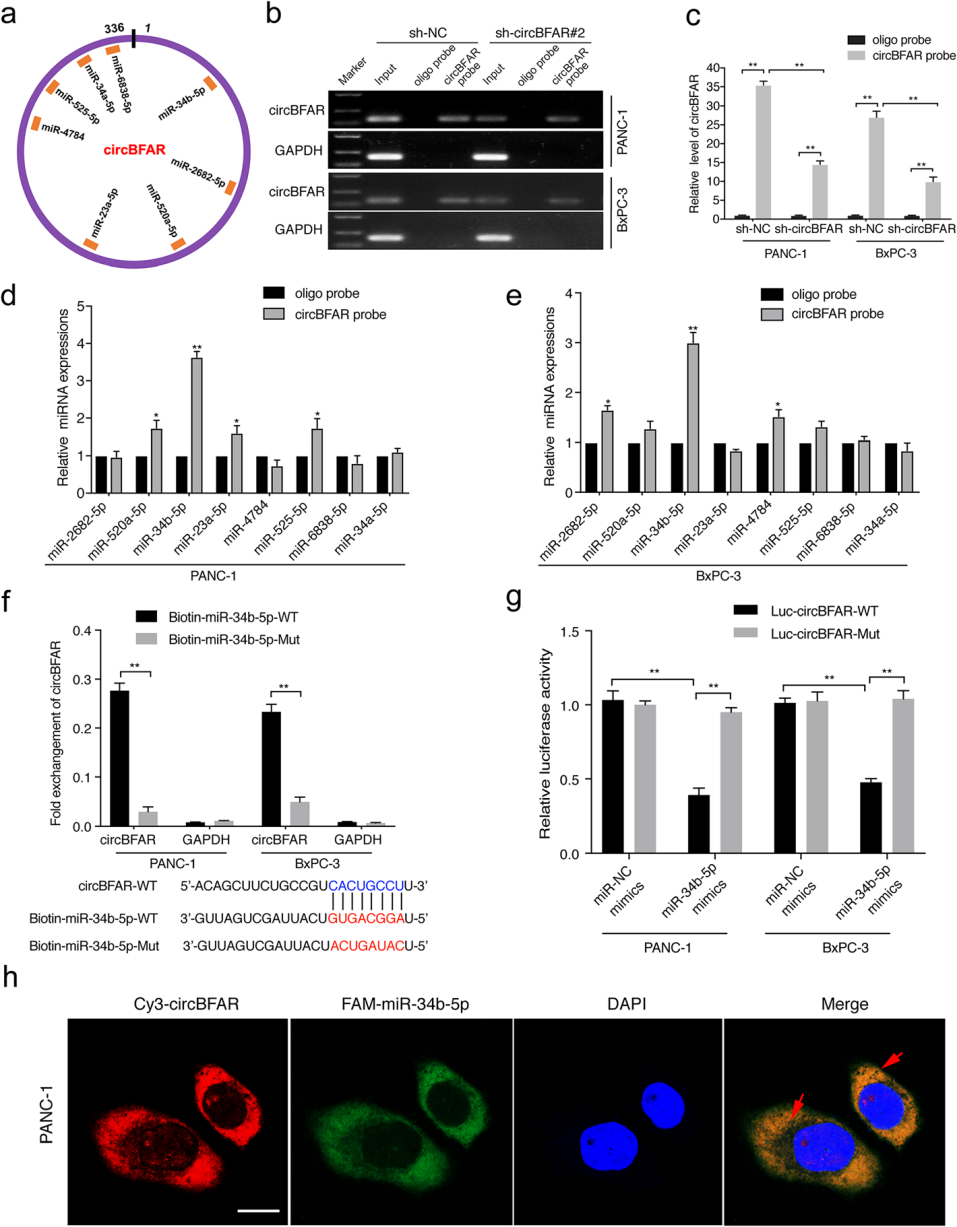
CircBFAR serves as a sponge for miR-34b-5p in PDAC cells. **a** Schematic illustration showing potential target miRNAs of circBFAR as predicted by miRanda, starbase, and RNA hybrid. **b**, **c** The specificity and efficiency of the circBFAR probe was validated using gel electrophoresis and qRT-PCR in PANC-1 and BxPC-3 cells. **d**, **e** qRT-PCR analysis of the expression of eight potential target miRNAs in PANC-1 and BxPC-3 cells. MiR-34b-5p was stably pulled down by circBFAR in PANC-1 and BxPC-3 cells. **f** Biotinylated miRNA pull-down (WT or mut) and qRT-PCR assays showing the expression levels of circBFAR after co-transfection of circBFAR and miR-34b-5p mimics in PANC-1 and BxPC-3 cells. GAPDH was used as the negative control. **g** The luciferase activities of the circBFAR luciferase reporter vector (WT or mut) measured after transfection with miR-34b-5p mimics or mimic NC into PANC-1 and BxPC-3 cells. **h** The co-localization of circBFAR and miR-34b-5p in PANC-1 cells was detected using a FISH assay. CircBFAR probes were labeled with Cy3. miR-34b-5p probes were labeled with FAM. Nuclei were stained with DAPI. The images were photographed at 1000X magnification. Scale bar = 10 μm. Statistical significance was assessed using two-tailed t-tests for two group comparison, and one-way ANOVA followed by Dunnett’s tests for multiple comparison. The error bars represent the standard deviations of three independent experiments. *P < 0.05, **P < 0.01

Previous studies showed that circRNAs mainly exhibited their functions in tumor progression by sponging to miRNAs [28, 29]. To determine which miRNAs interact with circBFAR, eight candidate miRNAs were predicted through overlapping the results of searching for MREs in the circBFAR sequence obtained from three public databases, miRanda (http://www.microrna.org), starbase (http://starbase.sysu.edu.cn/), and RNA hybrid (http://bibiserv.techfak.unibielefeld.de/rnahybrid/) (Fig. 4a). Next, we performed RNA pull-down assays to further confirm the binding partner of circBFAR. The specificity and efficiency of circBFAR probe were demonstrated by performing the RNA pull-down assays using a biotin-coupled circBFAR probe and an oligo negative probe in both shRNA-circBFAR and control vector transfected cells. The results showed that circBFAR was specifically enriched by the circBFAR probe and lower enrichment of circBFAR was observed in circBFAR knockdown PDAC cells (Fig. 4b-c). Pull-down assays using biotinylated probes specifically against circBFAR showed that miR-34b-5p was the only miRNA that specifically bound to circBFAR in PANC-1 and BxPC-3 cells (Fig. 4d-e). Moreover, an miRNA pull down assay with biotinylated wild-type (WT) and mutant (mut) miR-34b-5p showed that the miR-34b-5p-WT mimics captured more circBFAR than the biotinylated miR-34b-5p-mut, further confirming the interaction between circBFAR and miR-34b-5p (Fig. 4f). To validate the sponge effect of circBFAR, we applied a luciferase assay by co-transfection of miR-34b-5p mimics and a circBFAR-WT or a circBFAR-mut plasmid with a luciferase reporter into PANC-1 and BxPC-3 cells, respectively. The results showed that transfection of miR-34b-5p mimics significantly reduced the luciferase activity of the wild-type reporter, whereas no effect was shown by the mutant construct, indicating that circBFAR served as a miR-34b-5p sponge by binding to miR-34b-5p specifically at the CACUGCCU sites (Fig. 4g). Furthermore, FISH assays demonstrated that circBFAR and miR-34b-5p were co-localized in the cytoplasm of PDAC cells (Fig. 4h). In addition, we further investigate whether circBFAR affects the expression of miR-34b-5p. The qRT-PCR results showed that the expression of miR-34b-5p did not change after circBFAR knockdown in PANC-1 and BxPC-3 cells (Additional file 5: Figure S2b). Moreover, we found that no linear correlation was observed between the expression of circBFAR and miR-34b-5p in 208-case of PDAC tissues, further confirming that circBFAR interacted with miR-34b-5p without affecting the expression of miR-34b-5p (Additional file 5: Figure S2c). Taken together, our results indicated that circBFAR functions as a miR-34b-5p sponge in PDAC.

### miR-34b-5p inhibits proliferation, migration, and invasion of PDAC cells

**Fig. 5:**
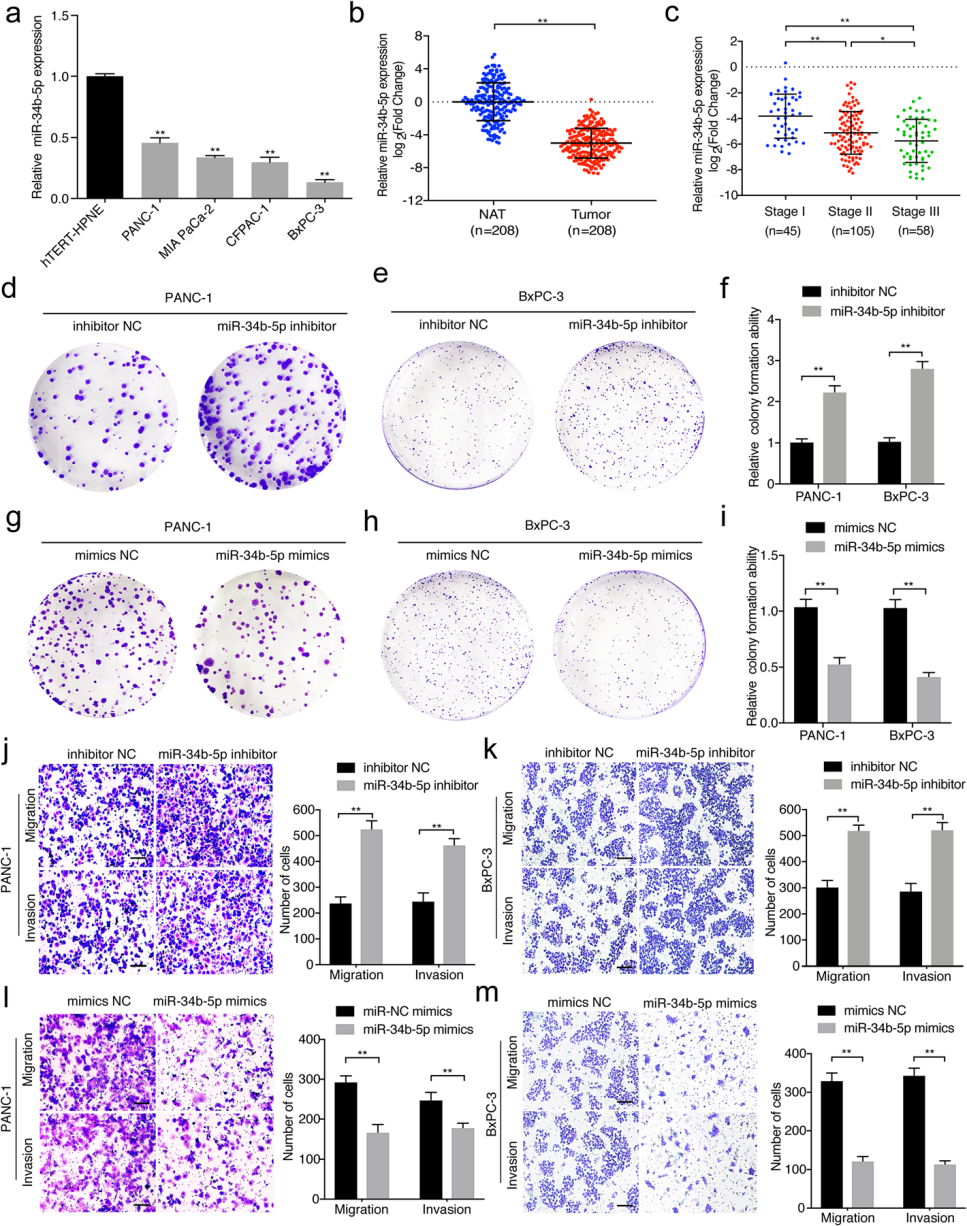
MiR-34b-5p inhibits the proliferation, migration, and invasion of PDAC cells. **a**, **b** qRT-PCR analysis of the relative expression levels of miR-34b-5p in pancreatic epithelial cells (hTERT-HPNE), PDAC cells (BxPC-3, MIA PaCa-2, CFPAC-1, and PANC-1) (**a**), and tissues (**b**). The nonparametric Mann-Whitney U-test was used. **c** The expression levels of miR-34b-5p in patients with PDAC with different pathological stages. **d-i** After transfection with miR-34b-5p mimics or inhibitor in PANC-1 and BxPC-3 cells, a colony formation assay was used to evaluate the colony formation ability of the cells. **j-m** Representative images of Transwell migration and Matrigel invasion assays showing the effect of migratory and invasive of PANC-1 and BxPC-3 cells transfected with miR-34b-5p mimics or inhibitors. The images were photographed at 100X magnification. Scale bar =100 μm. Statistical significance was assessed using two-tailed t-tests for two group comparison, and one-way ANOVA followed by Dunnett’s tests for multiple comparison. The error bars represent the standard deviations of three independent experiments. *P < 0.05, **P < 0.01

It has been reported that miR-34 acts as an anti-cancer role in various tumors [30, 31]. Thus, we further assessed the role of miR-34b-5p in PDAC. qRT-PCR analysis indicated that miR-34b-5p was significantly downregulated in PDAC cells compared with that in hTERT-HPNE cells (Fig. 5a). Consistently, analysis of a 208-case cohort of patients with PDAC showed that the expression of miR-34b-5p in PDAC tissues was lower than that in NATs and correlated negatively with tumor-node-metastasis (TNM) stage (Fig. 5b-c). To investigate whether miR-34b-5p functioned as an anti-cancer gene in PDAC, we further evaluated the function of miR-34b-5p in PDAC cells. Colony formation assays showed that miR-34b-5p knockdown significantly increased the proliferation of PDAC cells (Fig. 5d-f), while miR-34b-5p mimics obviously suppressed the colony formation ability of PDAC cells (Fig. 5g-i). Transwell assays demonstrated that the migration and invasion abilities of PDAC cells were also markedly enhanced after transfection with miR-34b-5p inhibitor (Fig. 5j-k). By contrast, the abilities of migration and invasion abilities of PDAC cells were inhibited by transfection with the miR-34b-5p mimics (Fig. 5l-m). Taken together, these results demonstrated that miR-34b-5p functions as a tumor suppressor and inhibits the proliferation, migration, and invasion of PDAC cells.

### MET is a downstream target of miR-34b-5p

**Fig. 6:**
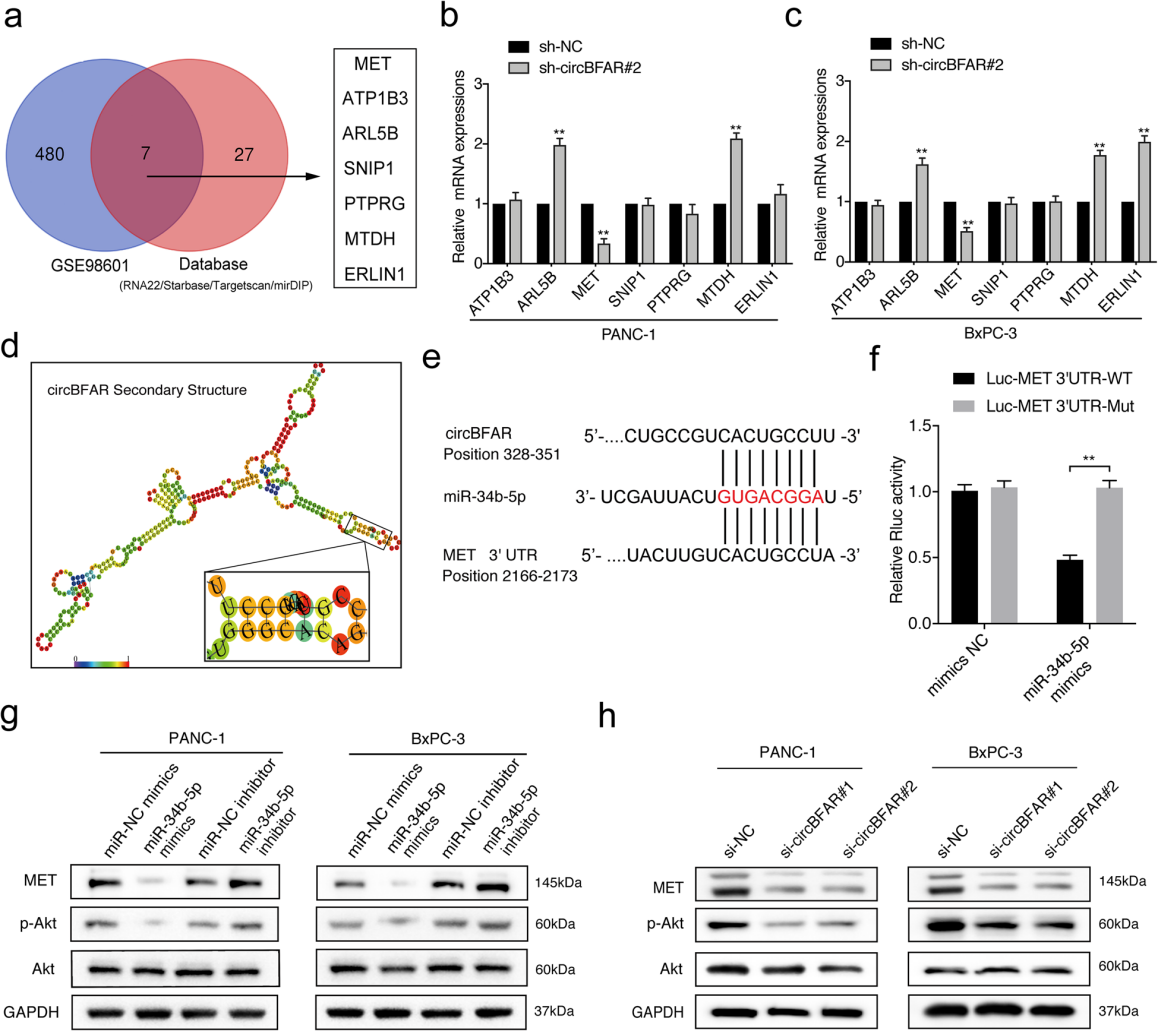
MET is a downstream target of miR-34b-5p. **a** Schematic illustration showing seven potential target genes of miR-34b-5p as predicted by the RNA22, Starbase, Targetscan, and mirDIP databases and the overlap with GSE98601. **b-c** qRT-PCR verified the expression of the predicted target genes in PANC-1 and BxPC-3 cells stably transfected with sh-circBFAR#2 and control shRNA. **d** Image showing the secondary structure of circBFAR and the possible binding sites with miR-34b-5p. **e** Matching sequence of miR-34b-5p with circBFAR and the 3′ UTR of MET. **f** The luciferase activities of the MET 3′ UTR luciferase reporter vector (WT or mut) were measured after transfection with miR-34b-5p mimics or mimic NC into PANC-1 cells. **g** Western blotting analysis protein levels of MET, Akt, and p-Akt (Ser 473) after transfection with miR-34-5p mimics or an inhibitor in PANC-1 and BxPC-3 cells. GAPDH was used as a loading control. **h** Western blotting analysis protein levels of MET, Akt, and p-Akt (Ser 473) after knocking down circBFAR in PANC-1 and BxPC-3 cells. GAPDH was used as a loading control. Statistical significance was assessed using two-tailed t-tests for two group comparison, and one-way ANOVA followed by Dunnett’s tests for multiple comparison. The error bars represent the standard deviations of three independent experiments. **P < 0.01

MiRNAs interact with the 3′ UTR region of target genes to regulate their expression. To further investigate the target genes of miR-34b-5p in PDAC cells, we analyzed microarray expression data (GSE98601) and the result indicated that 305 transcripts significantly changed upon the introduction of miR-34b-5p. Combined with the results predicted by software (RNA22, starbase, targetscan and mirDIP), we screened seven candidate genes for further validation (Fig. 6a). qRT-PCR showed that both miR-34b-5p overexpression and circBFAR knockdown dramatically decreased the expression of MET and upregulated the expression of ARL5B and MTDH in PDAC cells (Fig. 6b-c and Additional file 5: Figure S2d-e). To evaluate whether miR-34b-5p could directly bind to the 3′ UTR of MET, ARL5B and MTDH, we constructed luciferase reporter plasmids comprising the 3′ UTR of these genes. Luciferase reporter assays showed that miR-34b-5p mimics transfection downregulated the luciferase activity of MET 3’UTR but did not affect the luciferase activity of MTDH 3’UTR or ARL5B 3’UTR reporter, suggesting that miR-34b-5p regulated the 3’UTR of MET rather than ARL5B and MTDH. (Additional file 5: Figure S2f-h). Moreover, we found that circBFAR and the 3′ UTR region of MET shared the same sequence recognition sites that were complementary to miR-34b-5p, as predictedby Targetscan (http://www.targetscan.org/) and RegRNA2.0 (http://regrna2.mbc.nctu.edu.tw/), suggesting that MET might be the common downstream target of miR-34b-5p and circBFAR (Fig. 6d-e). Furthermore, we performed luciferase reporter assay with MET 3’UTR luciferase reporter plasmids (MET 3’UTR-mut) that containing mutated sequences of these recognition sites and the results showed that the luciferase activity was dramatically decreased after co-transfection of miR-34b-5p mimics and MET 3′ UTR-WT compared with that from MET 3′ UTR-mut, indicating that miR-34b-5p interacted with MET 3’UTR through recognition of theses sequences (Fig. 6f). Meanwhile, the results of western blotting further showed that both overexpression of miR-34b-5p and knockdown of circBFAR markedly reduced the level of MET and the level of its downstream signaling transducer, phosphorylated Akt (Ser 473), while there was no effect on the level of total Akt (Fig. 6g-h). Downregulating miR-34b-5p exhibited the opposite results (Fig. 6g). Collectively, these data suggested that miR-34b-5p could bind to the 3’UTR of MET to downregulate its expression.

### CircBFAR promotes PDAC proliferation, migration and invasion via the miR-34b-5p/MET axis

**Fig. 7:**
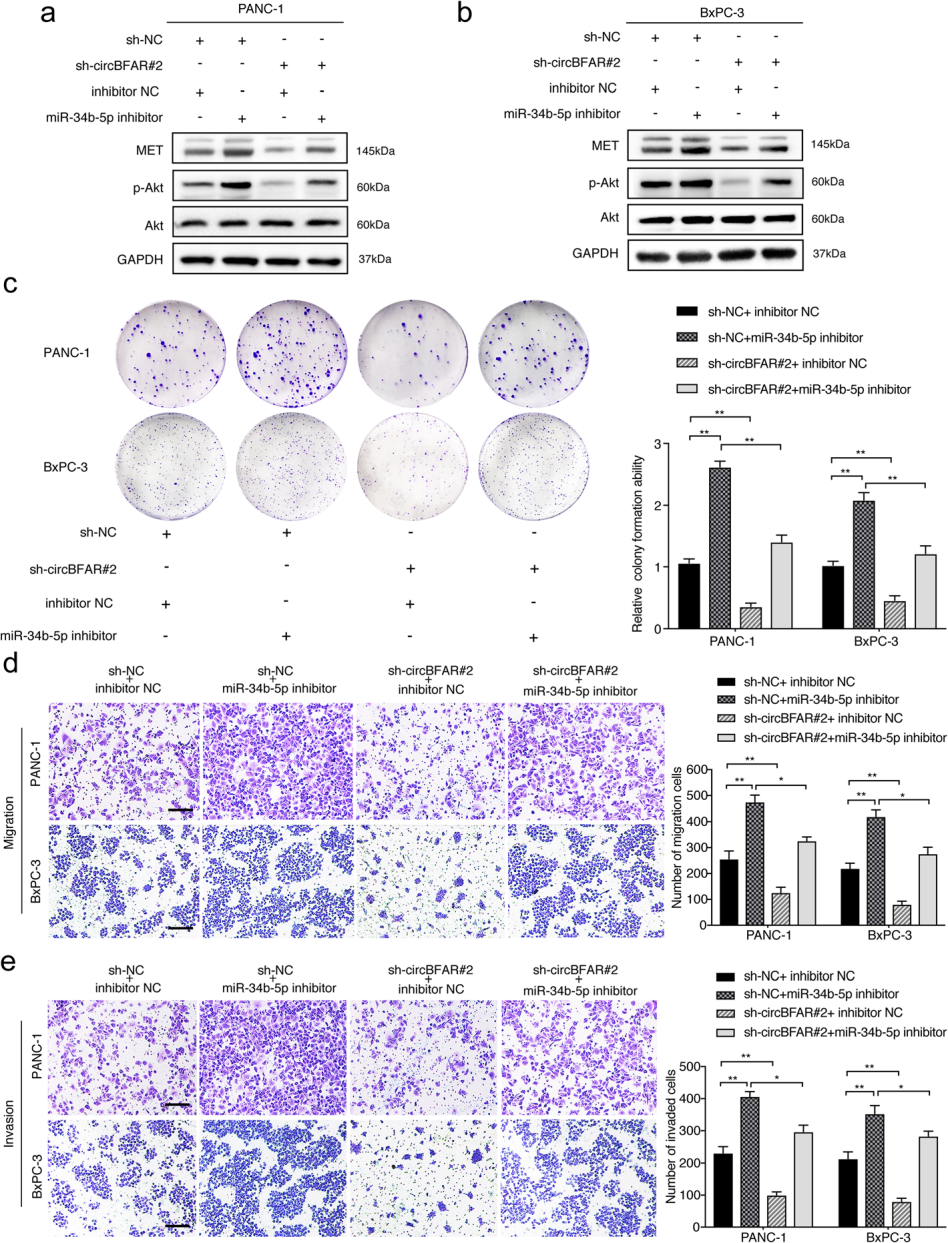
CircBFAR promotes PDAC proliferation, migration and invasion via the miR-34b-5p/MET axis. **a**, **b** The upregulation of MET and p-Akt (Ser 473), in PANC-1 and BxPC-3 cells transfected with the miR-34b-5p inhibitor was reversed by knockdown of circBFAR, as detected by western blotting. GAPDH was used as a loading control. **c-e** Colony formation, and Transwell migration and invasion assays demonstrated that transfection with the miR-34b-5p inhibitor increased the proliferation, migration, and invasion ability of PANC-1 and BxPC-3 cells; however, this function was reduced after co-transfection with sh-circBFAR#2. The images were photographed at 100X magnification. Scale bar = 100 μm. Statistical significance was assessed using two-tailed t-tests for two group comparison, and one-way ANOVA followed by Dunnett’s tests for multiple comparison. The error bars represent the standard deviations of three independent experiments. *P < 0.05, **P < 0.01

MET is a downstream target of miR-34b-5p and circBFAR; therefore, we further evaluated whether circBFAR upregulated MET through its role as a sponge of miR-34b-5p. Western blotting showed that silencing circBFAR attenuated MET expression and the phosphorylation of Akt (Ser 473), while this effect was abolished by co-transfection with an miR-34b-5p inhibitor (Fig. 7a-b). We next analyzed whether circBFAR-mediated sequestration of miR-34b-5p was responsible for the progression of PDAC. As demonstrated by colony formation experiments, knockdown of circBFAR inhibited the colony formation ability of PDAC cells, while the introduction of the miR-34b-5p inhibitor abolished this effect (Fig. 7c). Furthermore, circBFAR depletion significantly decreased the migration and invasion of PDAC cells, whereas this inhibition could be reversed by downregulation of miR-34b-5p (Fig. 7d-e). Taken together, these results indicated that circBFAR antagonizes miR-34b-5p-induced MET degradation and anti-cancer effects in PDAC.

**Fig. 8:**
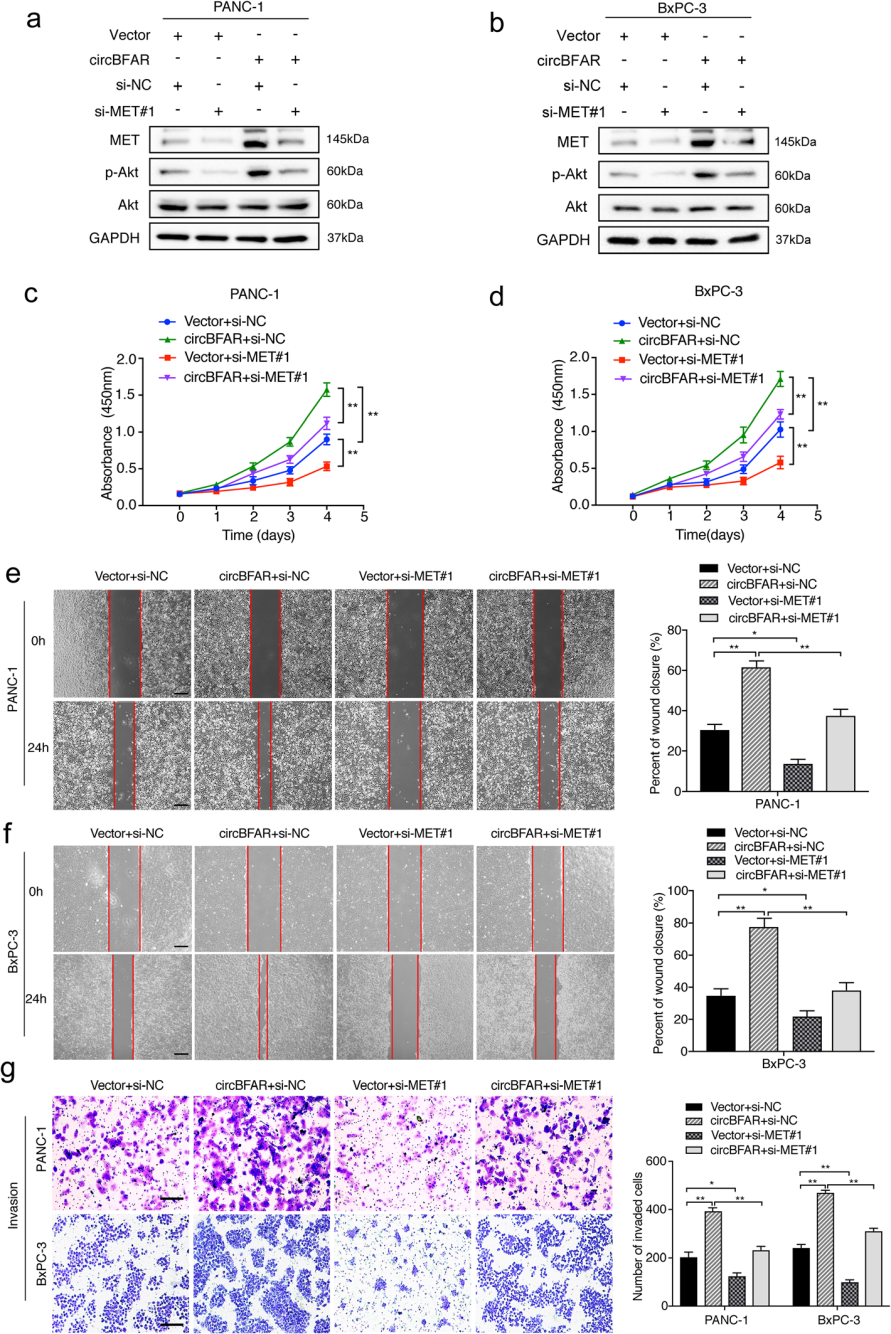
Downregulation of MET reverses the oncogenic phenotype induced by overexpression of circBFAR. **a**, **b** The upregulation of MET and p-Akt (Ser 473) in PANC-1 and BxPC-3 cells transfected with circBFAR was reversed by silencing MET, as detected using western blotting. GAPDH was used as a loading control. **c**, **d** CCK-8 experiments showing that transfection with circBFAR increased the proliferation ability of PANC-1 and BxPC-3 cells; however, this function was reversed after co-transfection with si-MET#1. **e**, **f**, **g** Wound healing, and Transwell invasion assays demonstrating that transfection with circBFAR increased the migration and invasion ability of PANC-1 and BxPC-3 cells; however, this function was reversed after co-transfection with si-MET#1. The images were photographed at 40X (**e**, **f**) or 100X (**g**) magnification. Scale bar =200 μm (**e**, **f**). Scale bar =100 μm (**g**). Statistical significance was assessed using two-tailed t-tests for two group comparison, and one-way ANOVA followed by Dunnett’s tests for multiple comparison. The error bars represent the standard deviations of three independent experiments. *P < 0.05, **P < 0.01

Next, we further verified whether the MET pathway was involved in circBFAR mediated progression of PDAC. Firstly, we constructed stable ectopic overexpression of circBFAR using a circBFAR plasmid in PDAC cells (Additional file 5: Figure S2i). Meanwhile, we analyzed the silencing efficiency of two short interfering RNAs targeting MET in PDAC cells and the results confirmed that the expression of MET was significantly downregulated in PDAC cells transfected with siRNAs. (Additional file 5: Figure S2j). Western blotting showed that circBFAR overexpression remarkably increased the level of phosphorylated Akt (Ser 473), while the effect was reversed after downregulating MET (Fig. 8a-b). Moreover, a cell proliferation assay demonstrated that overexpression of circBFAR promoted cell viability, while knockdown of MET reversed the circBFAR-induced proliferation of PDAC cells (Fig. 8c-d). Furthermore, both wound-healing and Transwell assays illustrated that overexpression of circBFAR significantly enhanced the migration and invasion of PDAC cells, whereas silencing MET attenuated this effect (Fig. 8e-g). Collectively, our results suggested that circBFAR promoted the progression of PDAC via the miR-34b-5p/MET axis.

### MET inhibitor reverses the oncogenic effect induced by overexpression of circBFAR in vivo

**Fig. 9:**
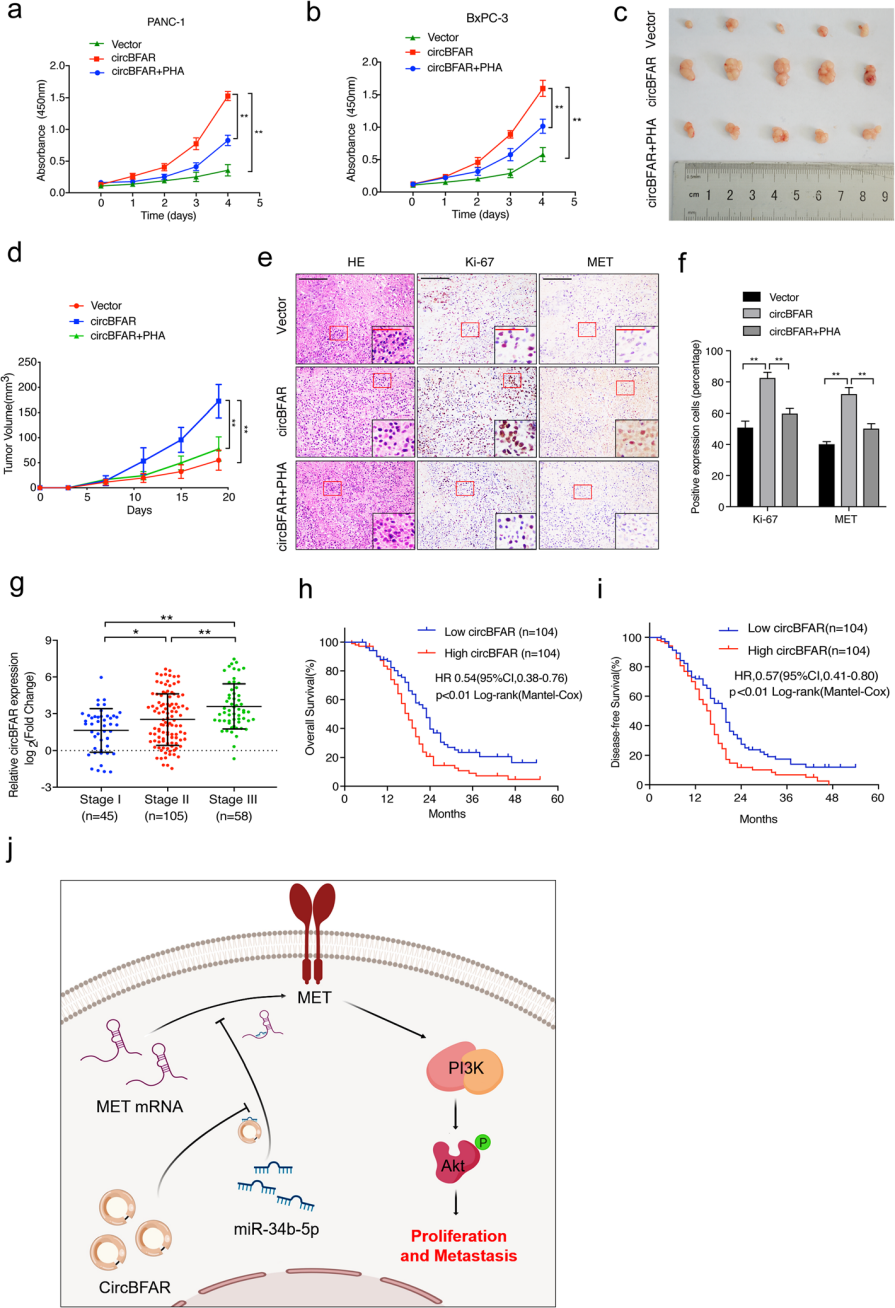
A MET inhibitor reverses the oncogenic effect induced by overexpression of circBFAR in vivo. **a**, **b** CCK-8 experiments showing that transfected circBFAR increased the proliferation ability of PANC-1 and BxPC-3 cells; however, this function was reversed after treatment with the MET inhibitor PHA. **c** Representative images of subcutaneous xenograft tumors. **d** The volume of the tumors increased markedly after treatment with circBFAR when compared with the vector group, while treatment with PHA attenuated this effect. **e**, **f** Representative HE and IHC staining images of subcutaneous tumors revealed the relative protein levels of Ki-67 and MET in the different groups. The images were photographed at 100X or 400X (insert) magnification. Scale bar: black =200 μm; red =50 μm. **g** qRT-PCR analysis the expression of circBFAR in PDAC tissues with different TNM stages. The nonparametric Mann-Whitney U-test was used. **h**, **i** Kaplan-Meier curves for OS (**h**) and DFS (**i**) of patients with PDAC with low vs. high expression of circBFAR. The median circBFAR expression was used as the cutoff value. **j** Schematic illustration showing the suggestion mechanism by which circBFAR promotes proliferation and metastasis in PDAC via the miR-34b-5p/MET axis. Statistical significance was assessed using two-tailed t-tests for two group comparison, and one-way ANOVA followed by Dunnett’s tests for multiple comparison. The error bars represent the standard deviations of three independent experiments. *P < 0.05, **P < 0.01

The application of inhibitors targeting the MET pathway have been tested in clinical trials for tumor intervention in recent years; therefore, we further examined whether blockage of MET signaling using an inhibitor was an effective strategy to reverse the oncogenic effect of circBFAR in PDAC. Overexpression of circBFAR promoted PDAC cell proliferation, while treatment with the MET inhibitor PHA-665752 (PHA) decreased the proliferation in circBFAR-transduced PDAC cells (Fig. 9a-b). To verify this effect in vivo, mice were divided into three groups and received subcutaneous injections of circBFAR-overexpressing or control PANC-1 cells to construct xenografts models. When the tumors reached 50 to 100 mm^3^ in size, the mice were received PHA or phosphate-buffered saline (PBS) treatment, respectively. The results showed that overexpression of circBFAR promoted tumor growth compared with that in the control groups; however, treatment with PHA attenuated the circBFAR-induced tumor progression (Fig. 9c-d and Additional file 5: Figure S2k). Moreover, IHC staining showed that the levels of MET and Ki67 were obviously increased by overexpression of circBFAR, whereas this effect was reversed upon treatment with PHA (Fig. 9e-f). These data demonstrated that administration of a MET inhibitor could significantly inhibit circBFAR-mediated tumorigenicity and proliferation of PDAC in vitro and in vivo.

### circBFAR overexpression correlates with poor prognosis of PDAC

To further investigate the clinical relevance of circBFAR in PDAC, we first correlated the expression of circBFAR with the clinicopathological characteristics in a 208-case cohort of patients with PDAC (Additional file 6: Table S3). We found that circBFAR overexpression correlated positively with TNM stage (Fig. 9g). Importantly, Kaplan-Meier analysis demonstrated that patients with PDAC with high circBFAR expression exhibited poor survival, including overall survival (OS) and disease-free survival (DFS), compared with those with low circBFAR expression (Fig. 9h-i). Moreover, the Cox proportional hazard model showed that circBFAR expression was an independent prognostic factor for OS and DFS in patients with PDAC (Additional file 7: Table S4 and Additional file 8: Table S5). Taken together, the clinical data suggested that circBFAR might serve as a potential biomarker for predicting the prognosis of patients with PDAC (Fig. 9j).

## Discussion

As novel RNA molecules, numerous recent investigations have demonstrated that circRNAs play important roles in pleiotropically modulated cellular function [32]. For instance, several circRNAs can function as miRNA sponges to regulate gene expression, combine with different proteins to influence function of associated proteins, or encode polypeptides that might have similar functions with their mRNA-encoded proteins [15, 33–35]. Although circRNAs have been demonstrated to be involved in the progression of multiple cancers, the precise mechanism of their impact on the biological processes of PDAC is largely unknown. In the present study, we screened for differentially expressed circRNAs in six pairs of PDAC and matched non-tumorous tissues and identified an uncharacterized circRNA, termed as circBFAR, which was highly expressed in PDAC. Gain and loss function experiments demonstrated that circBFAR promoted the tumorigenesis and aggressiveness of PDAC in vitro and in vivo. Moreover, circBFAR overexpression was closely related with poor prognosis of patients with PDAC. Mechanistically, we found that circBFAR upregulated MET expression via sponging miR-34b-5p; thereby further activating the MET signaling pathway to induce the proliferation and migration of PDAC. In addition, we demonstrated that inhibition of MET significantly inhibited circBFAR-induced tumorigenicity of PDAC in vivo. Thus, our findings identify a novel regulatory mechanism by which a circRNA promotes PDAC proliferation and metastasis, and provide a new strategy for PDAC treatment.

Accumulating evidence have revealed that circRNAs regulate cellular function as miRNA sponges. Thomas. et al. found that ciRS-7 functioned as a sponge of miR-7, resulting in increased levels of miR-7 targets [14]. Zheng. et al. reported that circHIPK3 acted as an miRNA sponge and bound to a host of miRNAs in human cancers [23]. Herein, RNA pull-down assays showed that circBFAR interacted with miR-34b-5p. Luciferase reporter assays validated the sponge effect of circBFAR on miR-34b-5p and further confirmed the binding sites on circBFAR. In addition, rescue experiments showed that the circBFAR knockdown-induced suppression of colony formation, migration, and invasion could be rescued using an miR-34b-5p inhibitor. Our results provided evidence to support the view that circBFAR binds to miR-34b-5p, acting as “miRNA sponge”, which is essential to the progression of PDAC.

Previous studies have reported that the miR-34 family mediates its antitumor effects in a variety of cancers [36]. Qu. et al. found that miR-34a was involved in long noncoding RNA lncARSR-mediated sunitinib drug resistance in renal cancer [37]. Chen. et al. provided direct evidence that mir-34 regulates the stem cell compartment by downregulating MET expression in prostate cancer [38]. Although miR-34 has been characterized as a vital tumor suppresser in tumor progression, the role and molecular mechanism underlying the regulation of miR-34b-5p in PDAC remains unclear. In the present study, we identified that miR-34b-5p directly bound to MET 3′ UTR to downregulate its expression. Moreover, MET depletion led to the inhibition of the MET signaling pathway, which ultimately resulted in the repression of PDAC progression. Importantly, we found that miR-34b-5p was sponged by circBFAR, which suppressed the targeting effect of miR-34b-5p on MET, leading to increased MET expression and the proliferation, migration, and invasion of PDAC. Therefore, the identification of the circBFAR/miR-34b-5p/MET axis expands our knowledge of the regulatory mechanism underlying PDAC progression.

Dysregulation of the MET signaling pathway occurs in a wide range of human cancers, including breast, colorectal, lung, pancreatic, hepatic, and ovarian cancers [39–42]. Inhibition of MET signaling has emerged as a promising approach for cancer therapy. Martinez-Marti et al. found that the combination of the MET inhibitor, capmatinib, and Erb-B2 receptor tyrosine kinases, ErbB-1/2/4, inhibitors successfully inhibited tumor growth in NSCLC-bearing mice [43]. Huang et al. reported that the combination of a MET inhibitor and an autophagy suppressor efficiently treated liver cancer in mice [44]. Despite the remarkable successes in animal experiments, most approved agents have proven insufficient to cure human patients. A lack of appropriate indicators is one of the important causes for this failure; therefore, developing biomarkers for MET-targeting therapy might represent an effective approach to improve its therapeutic efficiency in PDAC [45, 46]. In the present study, we found that overexpression of circBFAR significantly increased the expression of MET in PDAC. Moreover, silencing MET reversed circBFAR-induced progression of PDAC cells. Importantly, blockage of MET signaling using PHA dramatically inhibited the tumorigenesis in circBFAR-transduced PDAC-bearing mice in vivo*.* Our findings provide evidence to support circBFAR as a potential biomarker for clinical MET-targeting therapy in PDAC.

## Conclusions

In summary, we highlighted a new mechanism in which circBFAR aberrantly activates MET signaling by acting as a molecular sponge for miR-34b-5p, which subsequently promotes PDAC proliferation and metastasis. Our findings provide a novel insight into the mechanism underlying circRNA-induced progression of PDAC and could lead to the development of a potential biomarker and therapeutic target for PDAC therapy.

## Supplementary information

**Additional file 1: Table S1.** Patients’ background and characteristics.

**Additional file 2: Table S2.** The sequences of oligonucleotides and probes used in this study.

**Additional file 3.** Supplementary Methods.

**Additional file 4: Figure S1.** Silencing circBFAR inhibit proliferation, migration and invasion of PDAC cells in vitro.

**Additional file 5: Figure S2.** The identification and confirmation downstream target gene of miR-34b-5p and knockdown efficiency in PDAC cells.

**Additional file 6: Table S3.** Correlation between circBFAR expression and clinicopathologic characteristics of PDAC patients.

**Additional file 7: Table S4.** Univariate and multivariate analysis of Overall Survival for circBFAR expression in PDAC patients.

**Additional file 8: Table S5.** Univariate and multivariate analysis of Disease-free Survival for circBFAR expression in PDAC patients.

**Additional file 9: Table S6.** The sequences of primers used in this study.

**Additional file 10: Figure S3.** Full uncut original pictures.

## Data Availability

The microarray data of PDAC tissues and NATs analysed during this study are included in this published article (PMID: 27997903). The rest of datasets used and/or analysed during the current study are available from the corresponding author on reasonable request.

## Abbreviations

PDAC: Pancreatic ductal adenocarcinoma
circRNAs: circular RNAs
RBPs: RNA binding proteins
ANOVA: Analyses of variance
NATs: Normal adjacent tissues
FISH: Fluorescence in situ hybridization
EdU: 5-Ethynyl-2′-deoxyuridine
CCK-8: Cell counting kit 8
IHC: Immunohistochemistry
qRT-PCR: quantitative real-time PCR
SCID: Severe combined immunodeficient
HE: Hematoxylin and eosin
TNM: Tumor-node-metastasis
MET: Mesenchymal-epithelial transition factor
WT: Wild-type
mut: mutant
PHA: PHA-665752
PBS: Phosphate-buffered saline
OS: Overall survival
DFS: Disease-free survival
