# Supplementary material for: Circular RNA circBFAR promotes the progression of pancreatic ductal adenocarcinoma via the miR-34b-5p/MET/Akt axis
Source: Mol Cancer. 2020 May 6;19:83. doi: 10.1186/s12943-020-01196-4 (PMC7201986; doi:10.1186/s12943-020-01196-4)
Supplement: Supplementary file 1 — Additional file 1: Table S1. Patients’ background and characteristics. [file 12943_2020_1196_MOESM1_ESM.doc]

**Table S1. Patients’ background and characteristics.**

| **Characteristics** | **No. of cases (Percentage)** |
| --- | --- |
|
| **Total cases** | 208 |
| **Gender** |  |
| Male | 124 (59.6%) |
| Female | 84 (40.4%) |
| **Age, y** |  |
| ≤60 | 78 (37.5%) |
| ＞60 | 130 (62.5%) |
| **Body mass index, kg/m^2^** |  |
| <24 | 149 (71.6%) |
| ≥24 | 59 (28.4%) |
| **Smoking** |  |
| Never smoker | 124 (59.6%) |
| Past smoker | 25(12.0%) |
| Current smoker | 59(28.4%) |
| **CA19-9 level, IU/ml** |  |
| ＜35 | 21 (10.1%) |
| 35~10,000 | 145 (69.7%) |
| ＞10,000 | 42 (20.2%) |
| **CEA level, ng/ml** |  |
| ＜5 | 78 (37.5%) |
| ≥5 | 130 (62.5%) |
| **Tumor site** |  |
| Head of pancreas | 151 (72.6%) |
| Body/tail of pancreas | 57 (27.4%) |
| **Differentiation** |  |
| Poor | 39 (18.8%) |
| Moderate | 129 (62.0%) |
| Well | 40 (19.2%) |
| **T stage** |  |
| T1 | 51 (24.5%) |
| T2 | 59 (28.4%) |
| T3 | 79 (38.0%) |
| T4 | 19 (9.1%) |
| **Lymphatic metastasis** |  |
| Negative | 80 (38.5%) |
| Positive | 128 (61.5%) |
| **TNM stage** |  |
| Stage I | 45 (21.6%) |
| Stage II | 105 (50.5%) |
| Stage III | 58 (27.9%) |
| **Ki67 expression** |  |
| <5% | 10 (4.8%) |
| 6%~25% | 70 (33.7%) |
| 26%~50% | 73 (35.1%) |
| >50% | 55 (26.4%) |
| **Neoadjuvant chemotherapy** |  |
| No | 193 (92.8%) |
| Yes | 15 (7.2%) |
| **Adjuvant chemotherapy** |  |
| No | 19 (9.1%) |
| Yes | 189 (90.9%) |

Abbreviations: No. of cases = number of cases; T stage = tumor stage; TNM stage = tumor node

metastasis stage. ^A^ Chi-square test, ^*^ *p* <0.05, ^**^ *p* <0.01.
