# Supplementary material for: Circular RNA circBFAR promotes the progression of pancreatic ductal adenocarcinoma via the miR-34b-5p/MET/Akt axis
Source: Mol Cancer. 2020 May 6;19:83. doi: 10.1186/s12943-020-01196-4 (PMC7201986; doi:10.1186/s12943-020-01196-4)
Supplement: Supplementary file 2 — Additional file 2: Table S2. The sequences of oligonucleotides and probes used in this study. [file 12943_2020_1196_MOESM2_ESM.doc]

| **siRNAs** | |
| --- | --- |
| si-NC sense | UUCUCCGAACGUGUCACGUTT |
| si-NC antisense | ACGUGACACGUUCGGAGAATT |
| si-circBFAR#1 sense | CAGUAUUCUCCUCAGAUUATT |
| si-circBFAR#1 antisense | UAAUCUGAGGAGAAUACUGTT |
| si-circBFAR#2 sense | CUCCUCAGAUUAAUGAUGUTT |
| si-circBFAR#2 antisense | ACAUCAUUAAUCUGAGGAGTT |
| si-MET#1 sense | GUGUUGUAUGGUCAAUAACTT |
| si-MET#1 antisense | GUUAUUGACCAUACAACACTT |
| si-MET#2 sense | CGGAUAUCAGCGAUCUUCUTT |
| si-MET#2 antisense | AGAAGAUCGCUGAUAUCCGTT |
| **miR-34b-5p mimics and inhibitors** | |
| mimics NC sense | UUCUCCGAACGUGUCACGUTT |
| mimics NC antisense | ACGUGACACGUUCGGAGAATT |
| miR-34b-5p mimics sense | UAGGCAGUGUCAUUAGCUGAUUG |
| miR-34b-5p mimics antisense | AUCAGCUAAUGACACUGCCUAUU |
| inhibitor NC | CAGUACUUUUGUGUAGUACAA |
| miR-34b-5p inhibitor | CAAUCAGCUAAUGACACUGCCUA |
| **FISH Probes** | |
| Cy3-circBFAR | UGCUGCAAAACAUCAUUAAUCUGAGGAGA  AUACUGAC |
| FAM-hsa-miR-34b-5p | CAAUCAGCUAAUGACACUGCCUA |
| **Biotinylated probes** | |
| Biotin-circBFAR | UGCUGCAAAACAUCAUUAAUCUGAGGAGA  AUACUGAC |
| Biotin-miR-34b-5p | CAAUCAGCUAAUGACACUGCCUA |
| Biotin-miR-34b-5p mutant | GUUAGUCGUAAUGACACUGCCUA |

**Table S2. The sequences of oligonucleotides and probes used in this study.**
