# Supplementary material for: Circular RNA circBFAR promotes the progression of pancreatic ductal adenocarcinoma via the miR-34b-5p/MET/Akt axis
Source: Mol Cancer. 2020 May 6;19:83. doi: 10.1186/s12943-020-01196-4 (PMC7201986; doi:10.1186/s12943-020-01196-4)
Supplement: Supplementary file 3 — Additional file 3. Supplementary Methods. [file 12943_2020_1196_MOESM3_ESM.docx]

**Supplementary Methods**

**Cell culture**

Human PANC-1, MIA PaCa-2, BxPC-3, CFPAC-1, hTERT-HPNE and HEK-293 were purchased from the ATCC (American Type Culture Collection, Rockville, MD, USA). Cells were cultured with Dulbecco’s modified Eagle’s medium (DMEM, BI, Israel) or RPMI 1640 medium (BI, Israel) supplied with 10% fetal bovine serum (FBS, BI, Israel) and 1% penicillin/streptomycin and cultured at 37°C in humidified air with 5% CO_2_.

**Actinomycin D assay**

PANC-1 and BxPC-3 cells were seeded in a 6-well plate overnight and then treated with 2 mg/L actinomycin D (Sigma, USA) at indicated time. qRT-PCR assay assessed the stability of circBFAR after cancer cells were harvested. The experiments were performed three times.

**Fluorescence in situ hybridization**

The cell was collection and fix with paraformaldehyde after PANC-1 cells at 80–90% confluence and then pre-hybridized. At last, biotin-labeled circBFAR probe (Gene Pharma, China) hybridized with hybridization buffer at 37 °C overnight. All images were obtained with ZEISS confocal microscope (Carl Zeiss AG, Germany). The sequences of probes are listed in Additional file 2.

**CircRNA plasmid construction and stable transfection**

Stable knockdown of circBFAR was conducted through construct sh-RNA plasmid that purchase from Gene-Pharma (Shanghai, China). The human circBFAR and MET 3’UTR cDNA were synthesized by IGE (Guangzhou, China). circBFAR was cloned into pCD-ciR vector. The plasmids of MET 3’UTR and mutant luciferase reporters were synthesized by IGE (Guangzhou, China). After infect cells 48h with virus, then screened for 4–6 weeks with G418 (Life Technologies). The sequences of oligonucleotides are listed in Additional file 2.

**Oligonucleotide transfection**

For transient knockdown experiments, when cells were cultured to 60–70% confluence in 6-well plates, oligonucleotides purchased from Gene-Pharma (Shanghai, China) were transiently transfected using Lipofectamine RNAi Max (Invitrogen, USA) following the protocols. The sequences of oligonucleotides are listed in Additional file 2.

**RNA extraction and qRT-PCR**

Total RNAs of PDAC tissues and corresponding NATs or pancreatic cell lines were isolated with RNAiso Plus (TaKaRa, Japan) following the standard protocol. The total RNAs were reverse-transcribed as described by the manufacturer. qRT-PCR was performed to quantify the amount of circRNA, miRNA and mRNA, using TB Green" Premix Ex Taq^TM^ kit (Takara, Japan) and the Light Cycler 480 Detection System (Roche, Basel, Switzerland). GAPDH and U6 were used as internal control. The 2-∆∆CT method was used to calculate relative expression. The sequences of primers are listed in Additional file 9.

**5-Ethynyl-20-deoxyuridine (EdU) incorporation assay**

To assess the proliferation viability of cell, the EdU assay was carried out with a BeyoClick^TM^ EdU-555 detection kits (Beyotime, Shanghai, China). Transfected PDAC cells were seeded in twelve-well plates and incubated with complete medium for 12 h. After incubation with 50 mM EdU for 2 h, the cells were fixed and stained for 30 minutes. The nucleic acid was stained with Hoechst 33342. All images were captured with a fluorescent microscope.

**Colony formation assay**

For the colony formation assay, 500 transfected PDAC cells were plated into six-well plates and the cells were cultured in a humidified atmosphere containing 5% CO_2_ at 37 °C for 2 weeks. Then we washed cells two times with phosphate-buffered saline (PBS) and fixed cells with 4% paraformaldehyde for 20 min and then stained the cells with 0.1% crystal violet for 15 min. Visible colonies were then manually counted. Triplicate wells were measured for each treatment group and the record number was subjected to the statistical analysis.

**CCK-8 assay**

The proliferation of PANC-1 and BxPC-3 cells was assessed by CCK-8 kit (Beyotime, China) as the manufacturer’s instructions. About 48 hours after transfection, 2 x 10^3^ transfected PANC-1 or BxPC-3 cells were seeded in 96-well plates each well. We performed six independent replicates per treatment group. At 24, 48, 72 and 96 hours, 10% of CCK-8 reagent was added to each well and incubated at 37°C for 1.5 h. The absorbance of each well was measured at 450 nm with SPARK 10 M spectrophotometer (Tecan, Austria).

**Wound healing assay**

For wound healing assay, PDAC cells were seeded in six-well plates. The linearly scratch was made using 200μL sterile pipette tips when the cells adhered to the bottom of the plate. Cell migration patterns were captured at indicated time (0h and 24h) with an inverted microscope (Olympus Optical Co., Ltd., Tokyo, Japan). Migrated distance was measured and quantified.

**Transwell assays**

The migration and invasion abilities of PDAC cells were assessed by transwell assays. Cells were seeded into the upper chamber that was pre-treated with or without Matrigel (Matrigel BD biosciences, NY, USA) and each chamber loaded in 100μl serum-free culture medium and placed in 24-well tissue culture dishes. The lower chambers were filled with 600μl DMEM or 1640 containing 10% FBS. After 24 h of incubation, upper chamber cells were removed and invaded cells were fixed and stained. All images were captured with microscope (Olympus, Tokyo, Japan).

**Subcellular fractionation**

Total RNAs were extracted from cells using RNAiso Plus. Then cytoplasmic and nuclear fractions of cells were extracted using PARIS Kit (Life Technologies) according to the protocol. Then, the ratio of cytoplasmic and nuclear was measured by qRT-PCR. U6 served as the nuclear control, and GAPDH served as the cytoplasmic control.

**Biotin-labeled miRNA capture**

Firstly, biotinylated miRNA mimics or nonsense control which was synthesized by Gene-Pharma (Guangzhou, China), was transfected into PDAC cells with Lipofectamine RNAi Max (Life Technologies) and collected after 48h. At the same time, Streptavidin-Dyna Beads M-280 were washed and blocked on a rotator at 4 °C for 2 h. Then lysates mixed with blocked beads overnight at 4℃. The RNA was extracted and detected by qRT-PCR.

**Dual luciferase reporter assay**

For dual-luciferase assay, HEK-293T cells were seeded in 96-well plates at a density of 5 × 10^3^ cells per well before transfection and then were co-transfected with luciferase reporter plasmids and miR-34b-5p mimics. After 48 h transfection, Promega kit was used to detect luciferase activities of firefly and Renilla.

**Western Blotting**

Cells were harvested and lysed on ice in RIPA lysis buffer (CWBIO, China) with phosphatase and protease inhibitors (CWBIO, China) and followed by centrifugation at 14000 rpm for 20 min at 4 °C. Bicinchoninic acid (BCA) protein assay kit (CWBIO, China) was used to identified protein concentration. Proteins were separated by SDS-PAGE gels. Then proteins were transferred to PVDF membranes from SDS-PAGE gels. Then the membranes incubated with primary antibodies at 4 °C overnight and HRP-conjugated secondary antibodies. Followed by detection with ECL detection system (Millipore, Germany), and the images were captured using an Optimax X-ray Film Processor (Protec, Germany). The full uncut original pictures were show in Additional file 10.

**Immunohistochemistry (IHC)**

Formalin-fixed, paraffin-embedded PDAC tissues were dewaxed with 100% xylene and rehydrated with different graded ethanol. Briefly, the specimens were treated with sodium citrate in 121° C to retrieve antigen and cool down to room temperature. Then the slides were incubated with primary antibodies overnight at 4°C and then incubated with secondary antibodies at room temperature for 2 h. IHC stained were examined and scored independently by two observers for positively stained cells and immunohistochemical signal intensity.

**Microarray analysis**

CircRNA microarray is manufactured by Arraystar Technologies (Rockville, MD, USA). Each circRNA was accurately identified using a specific probe targeting the circRNA-specific junction. Differentially expressed circRNAs with statistical significance (fold changes ≥ 2 and *P* <0.05) between groups were identified using fold change cut-off. All of the differentially expressed circRNAs were annotated in detail according to the circRNA information. The microarray analysis work was performed by Yong Nuo Bio-tech, Guangzhou, People’s Republic of China.
