# Supplementary material for: Circular RNA circBFAR promotes the progression of pancreatic ductal adenocarcinoma via the miR-34b-5p/MET/Akt axis
Source: Mol Cancer. 2020 May 6;19:83. doi: 10.1186/s12943-020-01196-4 (PMC7201986; doi:10.1186/s12943-020-01196-4)
Supplement: Supplementary file 4 — Additional file 4: Figure S1. Silencing circBFAR inhibit proliferation, migration and invasion of PDAC cells in vitro. [file 12943_2020_1196_MOESM4_ESM.doc]

**
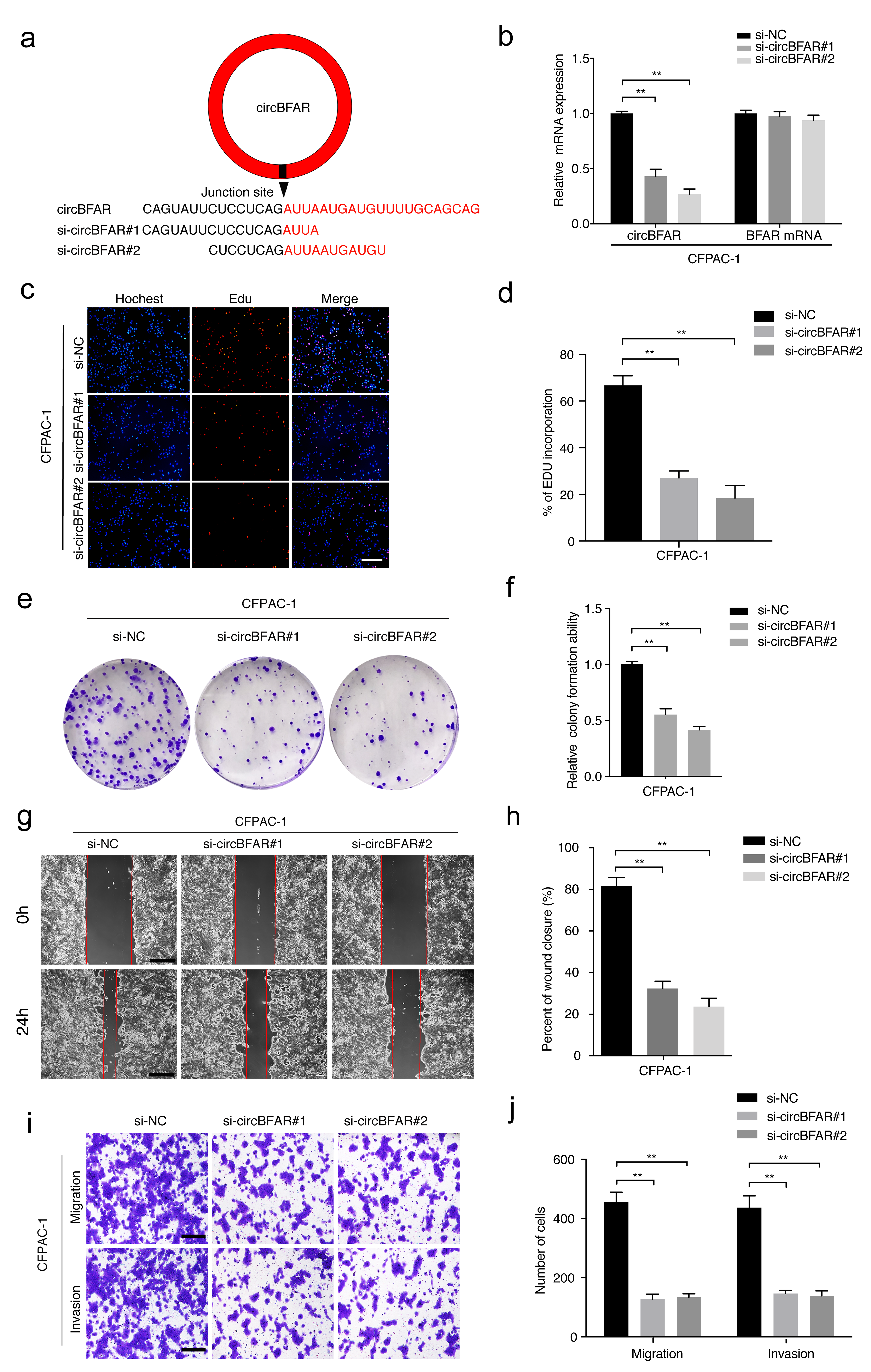
Additional file 4. Fig. S1 Silencing circBFAR inhibit proliferation, migration and invasion of PDAC cells in *vitro* a** Schematic illustration of two targeted siRNAs si-circBFAR#1 and si-circBFAR#2 targets the back-splice junction of circBFAR. **b** qRT-PCR analysis expression of circBFAR and BFAR mRNA in CFPAC-1 cells treated with si-circBFAR#1, si-circBFAR#2 or si-NC. **c, d** EdU assays showed the knockdown of circBFAR inhibited the DNA synthesis of CFPAC-1 cells. The images were photographed at 100X magnification. Scale bar = 100μm. **e, f** The cell proliferation ability was assessed by colony formation assay after knocking down circBFAR in CFPAC-1 cells. **g, h** The effect of circBFAR on cell migration capability was accessed by wound healing assay in CFPAC-1 cells treated with si-circBFAR#1 and si-circBFAR#2. The images were photographed at 40X magnification. Scale bar = 200μm. **i, j** The cell migration and invasion capabilities were assessed by transwell assay after knockdown of circBFAR in CFPAC-1 cells. The images were photographed at 100X magnification. Scale bar = 100μm. Statistical significance was assessed using two-tailed t-tests for two group comparison, and one-way ANOVA followed by Dunnett’s tests for multiple comparison. The error bars represent standard deviations of three independent experiments. **P* < 0.05, ***P* < 0.01.
