# Supplementary material for: Circular RNA circBFAR promotes the progression of pancreatic ductal adenocarcinoma via the miR-34b-5p/MET/Akt axis
Source: Mol Cancer. 2020 May 6;19:83. doi: 10.1186/s12943-020-01196-4 (PMC7201986; doi:10.1186/s12943-020-01196-4)
Supplement: Supplementary file 5 — Additional file 5: Figure S2. The identification and confirmation downstream target gene of miR-34b-5p and knockdown efficiency in PDAC cells. [file 12943_2020_1196_MOESM5_ESM.doc]

**
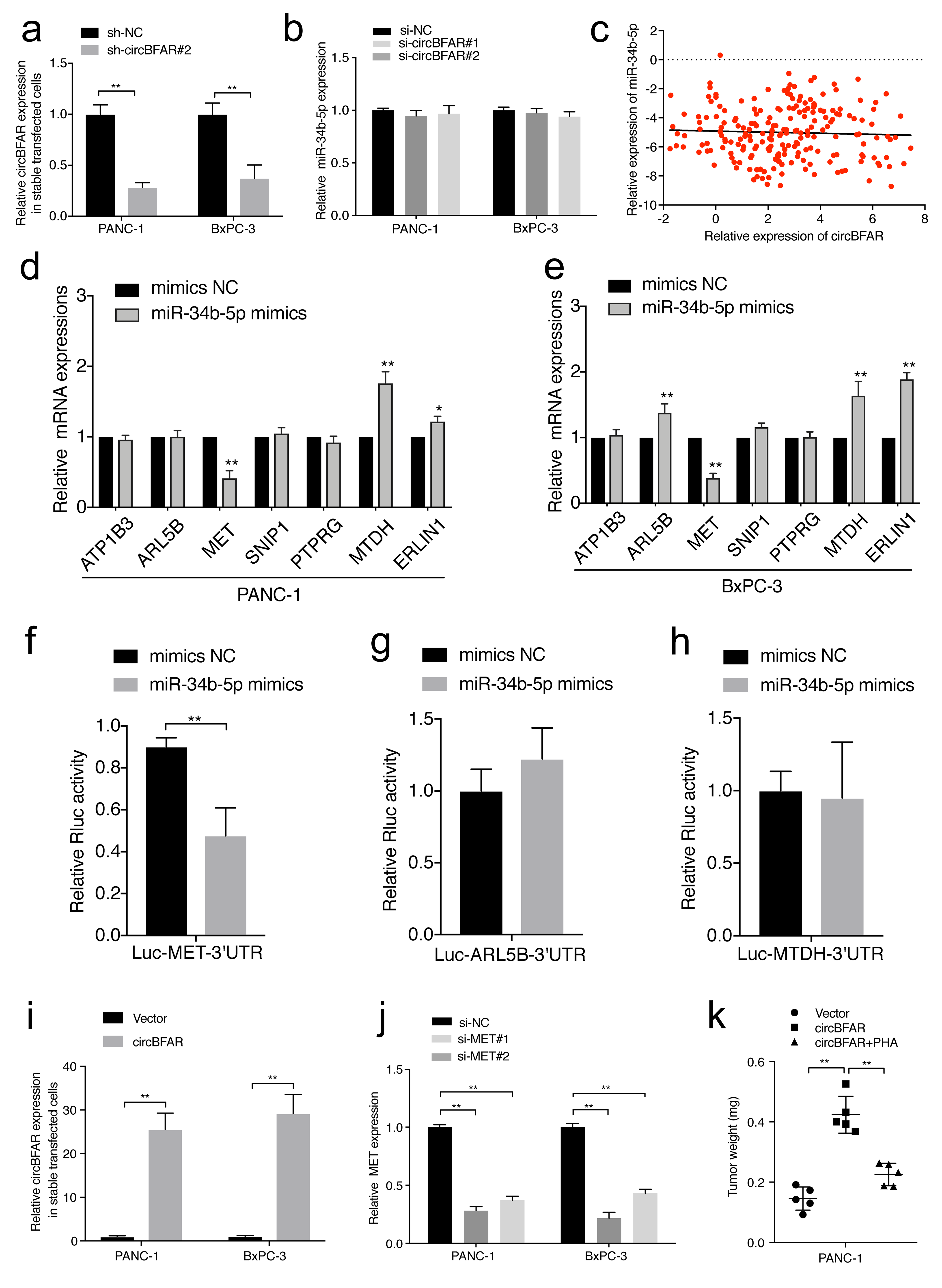
Additional file 5. Fig. S2 The identification of downstream target gene of miR-34b-5p and knockdown efficiency in PDAC cells. a** The knockdown efficiency of circBFAR was assessed by qRT-PCR in PANC-1 and BxPC-3 cells after transfected with sh-circBFAR plasmid and sh-NC plasmid. **b** qRT-PCR analysis expression of miR-34b-5p after knockdown of circBFAR in PANC-1 and BxPC-3 cells. **c** qRT-PCR analysis showed no linear correlation between circBFAR and miR-34b-5p in 208-case of PDAC tissues. **d,e** qRT-PCR verified the expression of predict target genes in PANC-1 and BxPC-3 cells that transfected miR-34b-5p mimics and control mimics. **f-h** Luciferase activity of MET 3’UTR, ARL5B 3’UTR and MTDH 3’UTR after co-transfection with miR-34b-5p mimics or mimics NC was assessed by luciferase reporter assays. **i** The overexpression efficiency of circBFAR was assessed by qRT-PCR in PANC-1 and BxPC-3 cells after stably transfected with circBFAR and vector. **j** qRT-PCR analysis expression of MET in PANC-1 and BxPC-3 cells treated with si-MET#1 and si-MET#2 and control cell as indicated. **k** The weight of the tumors increased markedly after treatment with circBFAR when compared with the vector group, while treatment with PHA attenuated this effect. Statistical significance was assessed using two-tailed t-tests for two group comparison, and one-way ANOVA followed by Dunnett’s tests for multiple comparison. The error bars represent standard deviations of three independent experiments. **P* < 0.05, ***P* < 0.01.
