# Supplementary material for: Circular RNA circBFAR promotes the progression of pancreatic ductal adenocarcinoma via the miR-34b-5p/MET/Akt axis
Source: Mol Cancer. 2020 May 6;19:83. doi: 10.1186/s12943-020-01196-4 (PMC7201986; doi:10.1186/s12943-020-01196-4)
Supplement: Supplementary file 6 — Additional file 6: Table S3. Correlation between circBFAR expression and clinicopathologic characteristics of PDAC patients. [file 12943_2020_1196_MOESM6_ESM.doc]

**Table S3. Correlation between circBFAR expression and clinicopathologic characteristics of PDAC** patients

| **Characteristics** | **No. of cases** | **circBFAR expression level** | | |
| --- | --- | --- | --- | --- |
|  |  | **Low** | **High** | ***p*-value^A^** |
| **Total cases** | 208 | 104 | 104 |  |
| **Gender** |  |  |  | 0.258 |
| Male | 124 | 66 | 58 |  |
| Female | 84 | 38 | 46 |  |
| **Age** |  |  |  | 0.390 |
| ≤60 | 78 | 42 | 36 |  |
| ＞60 | 130 | 62 | 68 |  |
| **Differentiation** |  |  |  | 0.659 |
| Poor | 39 | 17 | 22 |  |
| Moderate | 129 | 67 | 62 |  |
| Well | 40 | 20 | 20 |  |
| **T stage** |  |  |  | 0.578 |
| T1-2 | 110 | 57 | 53 |  |
| T3-4 | 98 | 47 | 51 |  |
| **Lymphatic metastasis** |  |  |  | 0.254 |
| Negative | 80 | 44 | 36 |  |
| Positive | 128 | 60 | 68 |  |
| **TNM stage** |  |  |  | **0.025^*^** |
| Stage I | 45 | 28 | 17 |  |
| Stage II | 105 | 55 | 50 |  |
| Stage III | 58 | 21 | 37 |  |

Abbreviations: No. of cases = number of cases; T stage = tumor stage; TNM stage = tumor node metastasis stage. ^A^ Chi-square test, ^*^ *p* <0.05, ^**^ *p* <0.01.
