# Supplementary material for: Circular RNA circBFAR promotes the progression of pancreatic ductal adenocarcinoma via the miR-34b-5p/MET/Akt axis
Source: Mol Cancer. 2020 May 6;19:83. doi: 10.1186/s12943-020-01196-4 (PMC7201986; doi:10.1186/s12943-020-01196-4)
Supplement: Supplementary file 7 — Additional file 7: Table S4. Univariate and multivariate analysis of Overall Survival for circBFAR expression in PDAC patients. [file 12943_2020_1196_MOESM7_ESM.doc]

**Table S4. Univariate and multivariate analysis of Overall Survival (OS) for circBFAR expression in PDAC patients (*n* = 208**)

| **Variables** | **Univariate analysis** | | | **Multivariate analysis** | | |
| --- | --- | --- | --- | --- | --- | --- |
|  | **HR** | **95%CI** | ***p*-value^A^** | **HR** | **95%CI** | ***p*-value^A^** |
| Gender (Female vs. Male) | 1.154 | 0.829-1.606 | 0.396 |  |  |  |
| Age (＞60 vs. ≤60) | 1.246 | 0.885-1.756 | 0.208 |  |  |  |
| Differentiation (poor and moderate vs. well) | 1.093 | 0.710-1.685 | 0.686 |  |  |  |
| T stage (T3-4 vs. T1-2) | 1.152 | 0.830-1.599 | 0.397 |  |  |  |
| Lymphatic metastasis (positive vs. negative) | 2.113 | 1.481-3.015 | **0.001^**^** | 2.068 | 1.448-2.953 | **0.001^**^** |
| circBFAR expression (High vs. Low) | 1.767 | 1.266-2.467 | **0.001^**^** | 1.718 | 1.228-2.402 | **0.002^**^** |

Abbreviations: HR = hazard ratio; 95%CI =95% confidence interval; T stage =tumor stage; TNM stage = tumor node metastasis stage. ^A^ Cox regression analysis, ^*^ *p* <0.05, ^**^ *p* <0.01.
