# Supplementary material for: Circular RNA circBFAR promotes the progression of pancreatic ductal adenocarcinoma via the miR-34b-5p/MET/Akt axis
Source: Mol Cancer. 2020 May 6;19:83. doi: 10.1186/s12943-020-01196-4 (PMC7201986; doi:10.1186/s12943-020-01196-4)
Supplement: Supplementary file 8 — Additional file 8: Table S5. Univariate and multivariate analysis of Disease-free Survival for circBFAR expression in PDAC patients. [file 12943_2020_1196_MOESM8_ESM.doc]

**Table S5. Univariate and multivariate analysis of Disease-free Survival (DFS) for circBFAR expression in PDAC patients (*n* = 208**)

| **Variables** | **Univariate analysis** | | | **Multivariate analysis** | | |
| --- | --- | --- | --- | --- | --- | --- |
|  | **HR** | **95%CI** | ***p*-value^A^** | **HR** | **95%CI** | ***p*-value^A^** |
| Gender (Female vs. Male) | 1.137 | 0.834-1.549 | 0.417 |  |  |  |
| Age (＞60 vs. ≤60) | 1.157 | 0.843-1.590 | 0.367 |  |  |  |
| Differentiation (poor and moderate vs. well) | 0.920 | 0.627-1.350 | 0.670 |  |  |  |
| T stage (T3-4 vs. T1-2) | 1.230 | 0.905-1.671 | 0.186 |  |  |  |
| Lymphatic metastasis (positive vs. negative) | 1.892 | 1.362-2.627 | **0.001^**^** | 1.856 | 1.335-2.580 | **0.001^**^** |
| circBFAR expression (High vs. Low) | 1.658 | 1.213-2.267 | **0.002^**^** | 1.620 | 1.183-2.217 | **0.003^**^** |

Abbreviations: HR = hazard ratio; 95%CI =95% confidence interval; T stage =tumor stage; TNM stage = tumor node metastasis stage. ^A^ Cox regression analysis, ^*^ *p* <0.05, ^**^ *p* <0.01.
