# Supplementary material for: Circular RNA circBFAR promotes the progression of pancreatic ductal adenocarcinoma via the miR-34b-5p/MET/Akt axis
Source: Mol Cancer. 2020 May 6;19:83. doi: 10.1186/s12943-020-01196-4 (PMC7201986; doi:10.1186/s12943-020-01196-4)
Supplement: Supplementary file 9 — Additional file 9: Table S6. The sequences of primers used in this study. [file 12943_2020_1196_MOESM9_ESM.doc]

**Table S6. The sequences of primers used in this** study.

| **Gene** | **Sequence (5’-3’)** |
| --- | --- |
| circBFAR-Forward | CTGCCTTGCTTTATGGTGGG |
| circBFAR-Reverse | ACTGCTGCAAAACATCATTAATCTG |
| linear BFAR-Forward | GGCCCTCAGATTTCTGTTAGTG |
| linear BFAR-Reverse | TGTGCCCACAGTTCAAGGTG |
| GAPDH-Forward | GGAGCGAGATCCCTCCAAAAT |
| GAPDH-Reverse | GGCTGTTGTCATACTTCTCATGG |
| divergent GAPDH-Forward | GAAGGTGAAGGTCGAGTC |
| divergent GAPDH-Reverse | GAAGATGGTGATGGGATTTC |
| ATP1B3-Forward | CCAAAATACCGTGACCAGATTCC |
| ATP1B3-Reverse | ACGAAGTTGGATCAGACCTACTG |
| MET-Forward | AGCAATGGGGAGTGTAAAGAGG |
| MET-Reverse | CCCAGTCTTGTACTCAGCAAC |
| ARL5B-Forward | GGGCTGATCTTCGCCAAACT |
| ARL5B-Reverse | CAGAGACTCCTGACCACCAAT |
| SNIP1-Forward | TGAAGCAGGAGCGTCTCAG |
| SNIP1-Reverse | TCGGTTTCTCTTACTGCGAGG |
| PTPRG-Forward | TGGAACCGTGTTGGTGGATTT |
| PTPRG-Reverse | CAACGTAGCCTTCTGTCAACG |
| MTDH-Forward | AAATGGGCGGACTGTTGAAGT |
| MTDH-Reverse | CTGTTTTGCACTGCTTTAGCAT |
| ERLIN1-Forward | TACTACAGGGGAGGAGCTTTAC |
| ERLIN1-Reverse | ACACTGCATAAGGAGCCAACA |
| hsa-miR-520a-5p | CUCCAGAGGGAAGUACUUUCU |
| hsa-miR-4784 | GCAAGCUUGAUGCAGCAUUACU |
| hsa-miR-23a-5p | GGGGUUCCUGGGGAUGGGAUUU |
| hsa-miR-34b-5p | UAGGCAGUGUCAUUAGCUGAUUG |
| hsa-miR-34a-5p | UGGCAGUGUCUUAGCUGGUUGU |
| hsa-miR-525-5p | AGGCAGUGUAUUGUUAGCUGGC |
| has-miR-2682-5p | UACGUUGCUGGAAGCUCCGGGU |
| has-miR-6838-5p | AGTGTGGCTTTCTTAGAGC |
| U6 | CTCGCTTCGGCAGCACA |
