# Supplementary figures and images for: Circular RNA circBFAR promotes the progression of pancreatic ductal adenocarcinoma via the miR-34b-5p/MET/Akt axis
Source: Mol Cancer. 2020 May 6;19:83. doi: 10.1186/s12943-020-01196-4 (PMC7201986; doi:10.1186/s12943-020-01196-4)

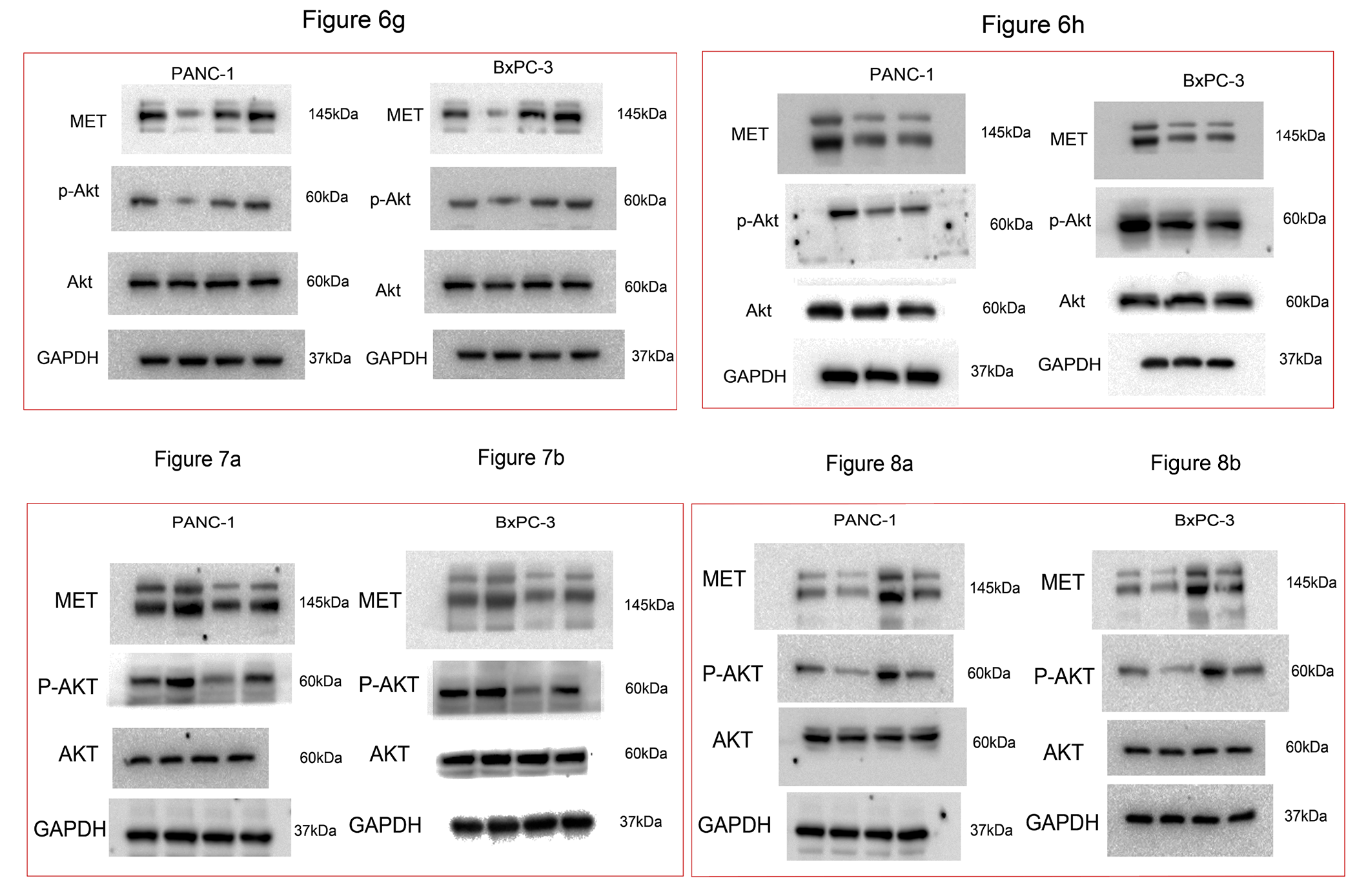


**Additional file 10. Fig. S3 Full uncut original pictures.**

Supplement: Supplementary file 10 — Additional file 10: Figure S3. Full uncut original pictures. [file 12943_2020_1196_MOESM10_ESM.doc]
